# Unveiling the Patterns of Wild Bee‐Plant Interactions on a Large and Mostly Unexplored Mediterranean Island (Sardinia, Italy)

**DOI:** 10.1002/ece3.73394

**Published:** 2026-04-06

**Authors:** Matteo Lezzeri, Vanessa Lozano, Giuseppe Brundu, Simone Flaminio, Ignazio Floris, Stephane Knoll, Michelina Pusceddu, Marino Quaranta, Carlo Polidori, Alberto Satta

**Affiliations:** ^1^ Department of Agricultural Sciences University of Sassari Sassari Italy; ^2^ National Biodiversity Future Center (NBFC) Palermo Italy; ^3^ CREA Research Centre for Agriculture and Environment (CREA‐AA) Bologna Italy; ^4^ Department of Environmental Science and Policy (ESP) University of Milan Milan Italy

**Keywords:** biodiversity, bipartite network, ecological networks, plant–pollinator interactions, protected area, wild bees

## Abstract

Plant–pollinator networks provide crucial insights into the mechanisms shaping the structure, stability, and ecosystem services of bee communities. Despite the Mediterranean basin being one of the world's richest regions for bee and plant diversity, it remains relatively understudied, with many islands largely overlooked in interaction studies. Here, we conducted an extensive survey of wild bees and their interactions with plants across four sites in Sardinia, the second–largest Mediterranean island, including two locations on Asinara Island with largely natural habitats and two sites in Nurra, a mainland area dominated by agroecosystems. In total, we recorded 122 wild bee species, including *Hoplitis corsaria* (Warncke, 1991), here reported for the first time from Italy, and six additional species newly recorded for Sardinia, highlighting the still incomplete exploration of the island's bee fauna. Bee diversity varied across sites, with higher richness at greater habitat heterogeneity, and at temporal scale, with consistent peaks of richness and diversity in May–July, likely according to variation in plant species richness but not in plant abundance. All bee–plant networks (divided by site and seasonal period) were highly specialized, modular, and non–nested, indicating low stability and potential vulnerability to disturbances. Network–level specialization, but not species–level specialization, varied among sites and also peaked in May–July, while marginally decreasing with plant species richness in the whole community. Since habitat heterogeneity was positively associated with percentage of both crop and urban cover, the observed patterns preliminarily suggested a role of anthropogenic disturbance on bee communities but not, or weakly, on network topology. Broader sampling across Sardinia will refine our understanding of habitat–driven effects.

## Graphical abstract

An extensive survey of wild bee and bee–plant networks in Sardinia (Italy) highlights high species richness and diversity, particularly in mainland agroecosystems. All interaction networks were highly specialized, modular, and not nested, indicating potential ecological vulnerability, though varying across sites and on a temporal scale. Site‐specific environmental conditions are preliminarily proposed as drivers of some of the observed differences in community traits and network structures.

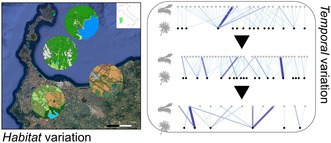

## 1 Introduction

Bees (Hymenoptera: Apoidea) represent one of the most tightly linked groups to flowering plants, as pollen constitutes the main food resource for larval development (Michener 2007). By collecting and transferring pollen among plant individuals, bees play a pivotal role in plant sexual reproduction (Potts et al. 2016; Ollerton 2017) and diversification processes (Van der Niet et al. 2014), while also sustaining key ecosystem services (Goulson et al. 2015; Klein et al. 2018). As a result, plant–pollinator interactions are recognized as one of the most important mutualistic relationships for the functioning of terrestrial ecosystems, underpinning the maintenance and diversity of the majority of angiosperms (Ollerton et al. 2011; Rodger et al. 2021), including a substantial fraction of global food crops (Klein et al. 2007; Gallai et al. 2009). Hence, conserving and maintaining diverse bee populations is of ecological, agricultural, and economic importance (Lautenbach et al. 2012; Potts et al. 2016; IPBES 2016; Klein et al. 2018).

Nevertheless, wild bees, together with other insect pollinators, have experienced widespread declines over recent decades, raising concern about an upcoming “pollinator crisis” (Potts et al. 2010; Burkle et al. 2013; Goulson et al. 2015; Ollerton 2017). Recent syntheses suggest that bee species richness has declined worldwide by 25% over a similar time span (Zattara and Aizen 2021). Additionally, many wild bee species have shown consistent population declines, accompanied by significant range contractions or shifts (e.g., Powney et al. 2019; Graham et al. 2021; Turley et al. 2022), with only a limited number of documented cases of range expansion (Gil‐Tapetado et al. 2024). These negative trends have been attributed to multiple, often interacting drivers including climate change, agrochemical use, pests and diseases, invasive species, and land use change (Goulson et al. 2015; Potts et al. 2016; IPBES 2016; Millard et al. 2021; Panziera et al. 2022). In particular, the long–term conversion of semi–natural habitats into intensive agricultural landscapes has resulted in severe losses of nesting sites and floral resources, strongly affecting wild bee communities (Le Féon et al. 2010; Kennedy et al. 2013; Nicholls and Altieri 2013; Goulson et al. 2015). However, the uneven geographical coverage of such studies—with Mediterranean areas particularly neglected—limits our ability to generalize patterns of pollinator decline and to understand how local environmental conditions, habitat heterogeneity, and resource availability jointly shape pollinator communities and their interactions with plants (Ghazoul 2015; Bartomeus et al. 2018; Herrera 2020; Millard et al. 2020, 2021; Wagner 2020; Wagner et al. 2021).

Hence, a key priority is the need for standardized and long–term monitoring of wild bees, particularly in data–deficient regions and along gradients of land use and anthropogenic pressure. Such monitoring is essential to improve our understanding of how pollinator communities respond to environmental change and to inform effective conservation and management strategies for pollinators, plants, and pollination services (Goulson et al. 2015; Potts et al. 2016; IPBES 2016; Wagner 2020; Graham et al. 2021; Halvorson et al. 2021; Millard et al. 2021).

Bipartite plant–pollinator networks provide a framework to explicitly describe how species interact within communities and how these interactions are organized across environmental contexts (Kaiser‐Bunbury and Blüthgen 2015; Harvey et al. 2017; Borchardt et al. 2021). By integrating information on species composition and interaction patterns, network approaches allow the assessment of key structural properties related to community organization, including interaction specialization, nestedness, and modularity, which have been linked to the stability and robustness of mutualistic systems under environmental change (Bartomeus et al. 2013; Burkle et al. 2013; Peralta et al. 2020; Borchardt et al. 2021; Turley et al. 2022; Payrató‐Borràs et al. 2024). As a result, plant–pollinator networks have become increasingly useful for comparing communities across habitat types and land–use gradients, and for evaluating how habitat alteration may affect pollinator–plant assemblages and their potential resilience to anthropogenic disturbances (Kaiser‐Bunbury and Blüthgen 2015; Harvey et al. 2017; Bascompte and Scheffer 2023). Despite this growing body of work, the application of network–based approaches remains uneven across regions. The Mediterranean basin, although widely recognized as a global hotspot for both bee diversity (Michener 1979; Winfree 2010; Nieto et al. 2014; Bartomeus et al. 2018; Nobile et al. 2021) and plant diversity (Comes 2004), is still underrepresented in plant–pollinator network studies, particularly in the southern European peninsulas (Iberia, Italy, and the Balkans) and on Mediterranean islands (e.g., Lanuza et al. 2025). Island systems, in particular, remain poorly explored despite their pronounced environmental gradients, strong seasonality, and sensitivity to both natural and anthropogenic disturbances (Sax and Gaines 2008), which make them especially suitable for investigating how local conditions and resource availability shape interaction networks (Traveset and Navarro 2018).

Islands are characterized by distinct ecological conditions in which species interactions are shaped by the interplay between local ecological filters and the size and composition of the regional species pool (Holt 1996; Traveset et al. 2016). As a consequence, plant–pollinator networks structure is expected to vary among islands, even when they are geographically close, reflecting differences in species richness, interaction opportunities, and environmental constraints (Neokosmidis et al. 2025; Wang et al. 2025). In particular, small islands often host fewer species and more simplified interaction networks with greater overlap among interaction partners, potentially increasing their vulnerability to pollination disruptions compared with larger islands (Traveset et al. 2016). The structure and stability of plant–pollinator networks are also strongly influenced by anthropogenic pressure, such as habitat loss and land–use intensification (Spiesman and Inouye 2013; Neokosmidis et al. 2025).

In this study, we investigate wild bee diversity and bee–plant interaction networks in north–western Sardinia, the second–largest island in the Mediterranean, at sites located on the main island and on a small satellite island and spanning contrasting land–use contexts, from semi–natural habitats to low–intensity agroecosystems. We specifically aimed to (1) describe in detail the wild bee communities and the bee–plant networks, (2) test for differences in communities and networks among sites and across seasonal periods, and (3) attempt to evaluate the potential roles of site–associated and period–associated floristic and land–use variation on the observed differences in communities and networks. By combining all these analyses, we ultimately aim to provide baseline information on wild bee communities and their interactions with flowering plants in a largely unexplored Mediterranean island system.

## 2 Materials and Methods

### 2.1 Study Area and Sampling Sites

**FIGURE 1:**
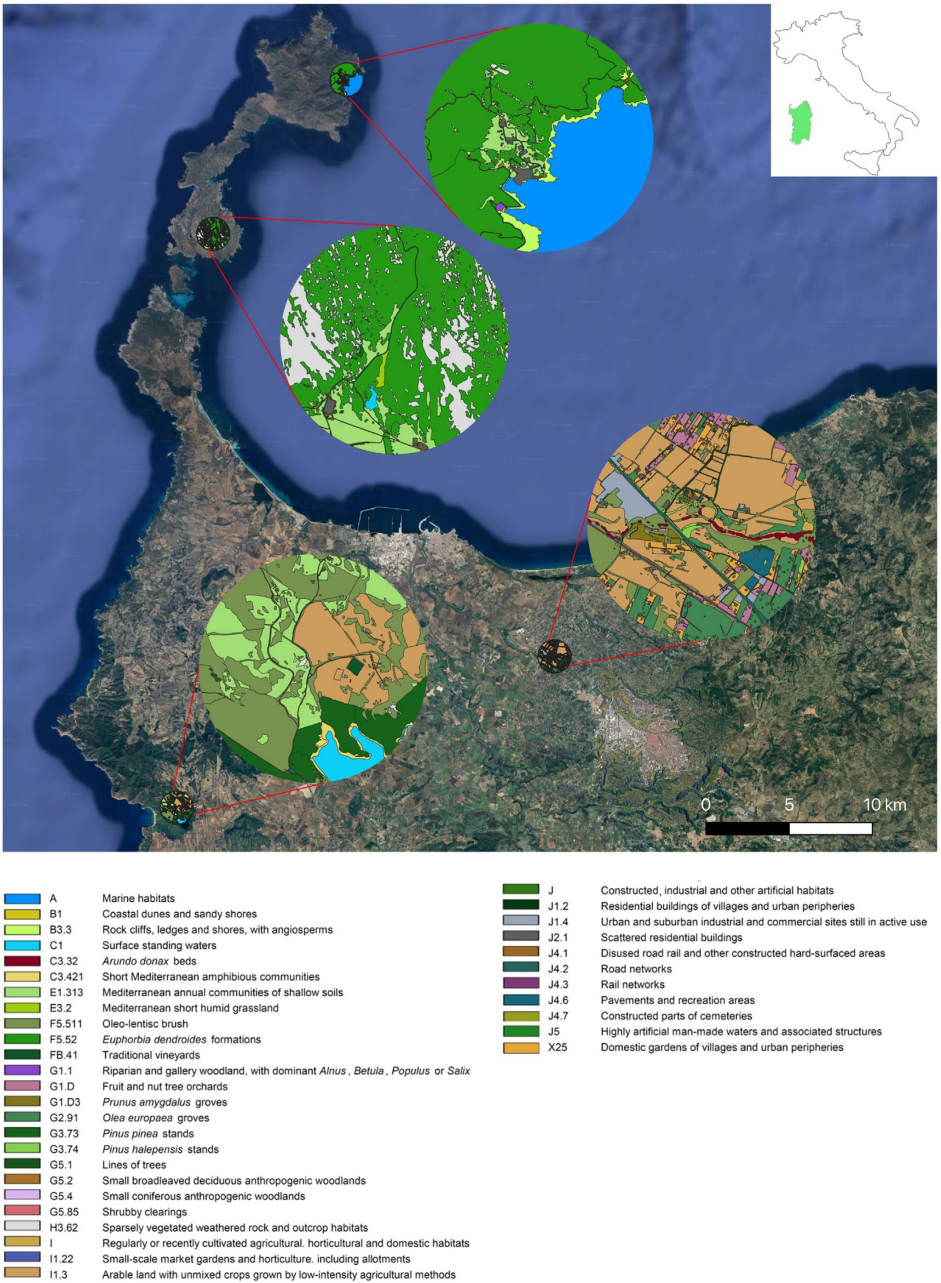
Landscape habitat composition within a 1 km radius around the sampling plots. For additional information about sampling sites, see Tables S1 and S2.

To identify and study wild bees' plant–pollinators networks, four sites in the north–western Sardinia were selected to represent contrasting landscape contexts in terms of habitat heterogeneity and degree of anthropization, allowing comparison of wild bee communities and interaction networks under different environmental conditions. Two sites were located on the island of Asinara (Fornelli and Cala d'Oliva) where a dominance of natural habitats characterizes the landscape, although many of the current plant communities still keep the legacy of grazing and past human activities (Treitler, Drissen, et al. 2017; Treitler, Buse, et al. 2017). Asinara is located at a sailing distance of < 2 nautical miles (≈3.7 km) from the Sardinian north–western coast and has a surface of 51.92 km^2^ (Figure 1). Cala d'Oliva sampling site is located in the northern part of the island of Asinara and is characterized by a scrub plant community with a prevalence of *Pistacia lentiscus* and *Euphorbia dendroides* . Fornelli sampling site is in the southern part of the island and is characterized by a scrub plant community with a prevalence of *P. lentiscus* , *Olea europaea* (wild type), and *E. dendroides* . Unlike the previous site, a higher presence of rock outcrops is observed, and the continuity with an area characterized by the presence of a Mediterranean temporary pond, between the transect and the road.

The two Asinara sampling sites can be considered representative of the same scrub plant community with evergreen sclerophyllous species, quite widespread throughout the island of Asinara and in other parts of Sardinia, partially degraded by grazing, and therefore with a significant presence or sometimes a dominance of the shrub *Cistus monspeliensis* , with herbaceous clearings with a prevalence of annual species. In the two sites, a few endemic species are present, although not particularly abundant, such as the Sardinian–Corsican endemic *Bryonia marmorata* in the southern site. The effects of grazing and browsing are very evident both on the soil and on the vegetation.

The other two sites were chosen on Sardinian mainland, in the Nurra region (Lago Baratz and Ottava), a flat area dominated by extensive agroecosystems with residual patches of natural vegetation. These mainland sites represent landscapes with a higher degree of land–use heterogeneity and anthropogenic influence compared with Asinara, thus enabling evaluation of how habitat composition and management context may be associated with differences in wild bee diversity and interaction structure. Baratz is one of the few natural lakes of Sardinia, as well as a Site of Community Importance (SCI). Lago Baratz sampling site is located about 1.5 km from the coast in a scrubland with a prevalence of *Olea europaea* (wild type), *Pistacia lentiscus* and few *Quercus ilex* trees. The transect crosses some hydromorphic areas, with a significant presence of the tree *Tamarix africana* , while in the more open and drier portions *Cistus monspeliensis* is common. Ottava transect is inside the experimental agricultural station “Mauro Deidda” and is in the immediate vicinity of diverse habitat types such as thermo–Mediterranean watercourses, areas with a high density of non–native species such as *Ailanthus altissima* , and anthropic elements such as roads, residential areas and small–scale livestock farms. Ottava sampling site is subject to occasional disturbs, which are however part of traditional low intensity agricultural practices, such as mowing and proximal transit of agricultural vehicles. Inside the experimental agricultural station there is a mosaic of agricultural crops, mainly annual crops, with the presence of one olive–groove, a small *Pinus halepensis* stand, and several windbreaks with different tree species (including non–native tree species).

In each sampling site, we estimated the total and percent cover of habitat types within a circular area of 1–km radius around each transect (Figure 1). This distance was considered large enough in relationship with average foraging ranges of wild bees (Gathmann and Tscharntke 2002). The habitat types in the four sampling sites were classified according to the EUNIS (European Nature Information System) habitat classification, developed by the European Topic Centre for Biodiversity for the European Environment Agency (EEA) (version 2012– available at: https://eunis.eea.europa.eu/habitats‐code‐browser.jsp; Chytrý et al. 2020). Google satellite imagery photointerpretation was used a basis for the identification and mapping of the habitat types in the four sampling sites, in a GIS environment (QGIS Development team 2023). For the subsequent statistical analyses, we then summed up, for each site, the % of all habitat types across each of the following categories: natural, semi–natural, natural/semi–natural, crop and urban, which were incorporated as explanatory variables in the comparative analyses of community and network patterns.

### 2.2 Bee Sampling

Wild bees were sampled monthly from February to November over the course of three consecutive years (2021, 2022, and 2023), using standardized transect walks (hereafter referred to as transects) (Westphal et al. 2008), corresponding to 10 sampling events per site per year. For that purpose, a permanent transect measuring 200 m in length and 2 m in width, divided into four equal 50‐m subunits (A–B, B–C, C–D, and D–C), was GPS located at each of the four sampling sites. The coordinates of the vertices of each subunit of the transect, and the average altitude of the surveyed areas, are reported in the Table S1.

Sampling occurred exclusively during optimal flight conditions for pollinators (i.e., temperature equal or above 15°C, wind strength lower than 3 on the Beaufort scale, and cloud cover not higher than 50%), for a fixed time length (15 min for each sub–unit of the transect), sampling twice a day during predetermined time slots (from 10:30 to 12:00 and 14:00 to 16:00) because not all bee species are active at the same time of the day (Pisanty et al. 2016). The surveillance period, recorded with a stopwatch, paused during specimen handling to allocate sufficient time to ensure an adequate transfer of bees into vials. The sampling process in the four transects was consistently executed by the same collector, within a maximum time interval of 5 days. All wild bee specimens sighted on flowers, except for easily recognizable ones such as *Bombus terrestris* , were captured using an entomological net and recorded alongside with the plant species they were visiting at the time of capture (Lezzeri et al. 2024). All bee specimens were identified by three expert taxonomists (SF, MQ, and ML) through morphological analysis, using dichotomous keys and comparison with the Research Centre for Agriculture and Environment reference collection. All visited plant species were identified by VL and GB using dichotomous botanical keys and field expertise. Given the absence of *Apis mellifera* at the Asinara sites (Lezzeri et al. 2024) and the low density of managed hives at the Nurra mainland sites (Italian National Beekeeping Registry, BDA), and to specifically focus on wild bee communities and their interactions with flowering plant, honey bees was excluded from transect counts.

### 2.3 Floristic Surveys

Floristic surveys were conducted concurrently with wild bee sampling, in each subsection (AB, BC, CD, and D–C) of the four transects. Importantly, the floristic survey was carried out considering only tracheophytes with entomophilous pollination, and with single flowers (or inflorescences) larger than 0.5 cm. To determine the relative abundance of a plant species of interest for pollinators in each subsection, we visually assessed the total flower cover (of all species summed) of a transect and then the relative proportion of each plant species to the total cover. A 3‐year survey is likely to provide an overview of most of the species present much better than a single–year survey, due to the typical temporal variability of grassland plant communities (e.g., Fernández‐Moya et al. 2011). The nomenclature for native plants follows Bartolucci et al. (2024) and Galasso et al. (2024) for non–native plants. Floristic richness was estimated by counting only the number of flowering species in each transect monthly. The data was then pooled across years due to similarities in the floristic composition. The raw data are available in the Supporting Information S1—DATASET_FlorSurv.xlsx.

### 2.4 Bee and Plant Species Diversity

First, we calculated Chao 1, which is a non–parametric estimator (distribution–free) of total bee species richness (Chao 1984), using the whole dataset (all periods and years pooled), in order to evaluate to which extent we covered the likely true species richness of bees of the studied communities. By using the number of rare species that are found in a sample, this index calculates the likelihood of the presence of unsampled species, thus suggesting how much complete were the samplings (Chao 1987). Furthermore, the iNEXT package in R software v.4.2.2 (R Core Team 2021) was used to inspect rarefaction and extrapolation curves for bee diversity, based on cumulative incidence of species across plots, months and years.

We described community diversity by calculating commonly used indices which take into account both the richness (number of sampled species) (*S*) and the abundance of species (number of individuals belonging to each sampled species) (*N*). All indices were calculated for bee communities recorded in the transects, for plant communities recorded in the transects, and for plant communities as recorded in the floristic surveys, in all cases by first pooling the data across the 3 years and then dividing them *per* periods spanning 3 or 4 months (February–April, May–July, August–November). Calculations were then done on datasets separated by sites and period.

The Shannon–Wiener diversity (*H*) measures diversity by calculating uncertainty about the identity of species in the sample, and its units quantify information (Shannon and Weaver 1949). The Gini–Simpson index (*GS*, i.e., 1–Simpson index) calculates dominance by measuring the probability that two individuals, drawn randomly from the sample, will be of different species (i.e., the lower the index, the higher the dominance) (Simpson 1949). For plant species in the community (floristic surveys), the abundance of each species was calculated by the average % cover across transect subsections and years and period, and then the sum of all species' abundances was used as an estimate of overall abundance (i.e., whole flower cover) *per* site and period. Given the different phenology of the plants at the four sites (as expressed in variation of maximum number of species/plot/year) (see Figure S1), three periods were used for Baratz and Ottava and two periods were used for Cala d'Oliva and Fornelli (where almost no flowering plants were recorded after July). The software PAST version 4.17 (Hammer et al. 2001) was used for all these calculations.

### 2.5 Bee–Plant Networks

Also for interactions, data were pooled from all transect subunits and across 3 years of sampling, while then dividing them by period and site as above, for a total of 10 pollination networks. Each network was depicted as a matrix, with rows corresponding to flowering plant species (n) and columns to bee species (m); each cell indicates the number of interactions. The data matrices are provided in the Supporting Information S1—DATASET_BeePlant.xlsx.

The bee–plant networks were built and analyzed with the “*bipartite*” package (Dormann et al. 2008) in R software v.4.2.2. This package represents the networks as bipartite graphs and calculates different quantitative descriptors for the networks (Blüthgen et al. 2006, 2008; Dormann et al. 2008, 2009). In this study, the following quantitative indices have been calculated, the first three at network–level and the last one at species–level:

- *Complementary specialization* (*H*_*2*_ *′*). This index measures how well specialized interactions among species complement each other to form a balanced and integrated network, reflecting the degree of specialization within the network. *H*_*2*_ *′* ranges from 0 to 1, with high specialization (closer to 1) indicating high dependency of each species on a few exclusive partners and low specialization (closer to 0) indicating higher functional redundancy. *H*_*2*_ *′* is not affected much by variation in sampling effort and by the incompleteness of sampled plant–pollinator interactions (Blüthgen et al. 2006).
- *Weighted nestedness* (*WNODF*). This index assesses whether species with few partners (specialist species) interact with a subset of the partners that more generalist species interact with, taking into account the frequency of plant–pollinator interactions. *WNODF* ranges from 100 (perfect nestedness) to 0 (randomly distributed interactions). Greater nestedness confers higher stability in mutualistic networks.
- *Modularity Q* (*M*). This index measures how sets of interacting species aggregate in modules, these defined by more prevalent interactions than those between modules. *M* varies between −1 and 1, with positive values indicating a modular structure, 0 indicating no modularity, and negative values indicating less modularity than random.
- *Bee or plant specialization* (*d'*). This index is calculated at species level, both for bees and plants in the networks, and measures the extent to which a single species' interactions are specialized relative to the availability of partners within the network. *d'* ranges from 0 to 1, where 0 indicates complete generalization and 1 indicates complete specialization. *d'* values give insights on competition for resources (pollinators) or services (plants) between species in the network. As for *H*_*2*_ *′*, *d'* is not heavily affected by sampling effort (Blüthgen et al. 2006; Polidori et al. 2013).

### 2.6 Statistical Analysis

To verify if bee and plant communities in the networks differed across sites and periods, we calculated the 95% confidence intervals of community diversity parameters (*S*, *H*, *GS*) as well as for the abundances (*N*) with a bootstrap procedure (9999 random samples). Then, comparisons between the observed and the bootstrapped differences in the diversity indices were made to determine if the former are statistically significant. This analysis was carried out in PAST version 4.17. To verify if *H*_*2*_ *′*, *WNODF* and *M* differ to what one would expect under random networks (2 replicates) built using the same observed row and column totals of the matrices, we compared the observed value with a null distribution obtained from 1000 networks generated randomly (with the r2dtable algorithm), and subsequently calculating the *z*–score (= (observed value—mean of null model values)/standard deviation of null model values). The index is significantly different from expected when the |*z*–score| is > 1.96. Since the number of networks per site and period was limited, it was not possible to apply inferential statistical tests to test if *H*_*2*_ *′*, *WNODF* and *M* differed across networks. Hence, differences between networks were assessed descriptively by comparing the standardized values (*z*–scores) of the network indices obtained with respect to the null models. These analyses were performed using the package “*bipartite*” in R software v.4.2.2.

To test for possible effects of plant community traits (from floral surveys) (*S*, *N*, *H*, *GS*) and of habitat traits (habitat heterogeneity (Shannon diversity of land–use types), % natural cover, % semi–natural cover, % natural/semi–natural cover, % crop cover and % urban cover) on the variation of bee and visited plant communities, of network–level indices and of species–level indices (the latter for both bees and visited plants), we carried out a series of models. Given the large number of the floristic and habitat explanatory variables (10), a correlation matrix was first performed to avoid including highly correlated variables in the models. The correlations indicate that plant (community) *S* was correlated with plant (community) *H* and *GS*, but not with abundance *N* (Figure S2), so that *S* and *N* were chosen among this set of variables. On the other hand, habitat heterogeneity *H* was correlated with % natural cover, % semi–natural cover, % natural/semi–natural cover, % crop cover and % urban cover (Figure S2). Hence, we only enter habitat heterogeneity *H*, which was also not correlated with plant (community) *S* and only weakly with plant (community) *N*, in the models.

For the models including the 10 networks as cases (i.e., those with community–level and network–level indices as dependent variables), we only could perform individual linear models, since the sample size was too low to allow including more than one explanatory variables in the models. For species–level indices, models included much more cases since *d'* is a unique value for each species. Hence, in these cases, we carry out generalized linear models (GLMs) in which a number of floristic and habitat traits, as well as site and period and their interactions, were included as explanatory variables. We did not include the number of individuals per species as a further co–variable since *d'* was not correlated with it in either bees (*R* ^2^ = 0.0047) or plants (*R* ^2^ = 0.0049). For the species–level models, the overall effects of period, site and their interactions were shown following an ANOVA performed on the model, since no paired differences between sites, periods or interactions were detected in the GLMs. A Gaussian family was used in the models in case of continuous dependent variables, while for dependent variables based on count data (*S*), a Poisson family was applied. All these analyses were performed using the function lm(), glm() and aov() in R software v.4.2.2.

## 3 Results

### 3.1 Habitat Diversity

The landscape analysis revealed a high degree of habitat heterogeneity across the four sampling sites, with a total of 36 distinct habitats and land–use types identified. These included 14 natural and semi–natural habitats, 11 urban habitat types characterized by high anthropogenic pressure, and 11 agricultural land–use types, mainly low–intensity arable systems (Figure 1, Table S2). On the island of Asinara, the two sites (Cala d'Oliva and Fornelli) were dominated by semi–natural habitats, covering over 60% of the total area at both locations, particularly *Euphorbia dendroides* formations. Agricultural areas were almost completely absent (Figure 1, Table S2). In the Nurra region, Ottava was dominated by cultivated areas (mainly arable land with mixed crops as low–intensity agricultural methods), covering more than 65% of the total area. Semi–natural habitats were also represented, comprising about 19% of the total area. Additionally, a significant presence of urban habitats, accounting for nearly 16% of the total area, was detected in Ottava. These urban habitats mainly included commercial and industrial sites, as well as domestic gardens in villages and urban suburbs. In contrast, at the Baratz site, semi–natural habitats (primarily Oleo–lentisc brush and Mediterranean annual communities of shallow soils) covered about 57% of the total area, compared to 24% for cultivated areas (Figure 1, Table S2).

### 3.2 Bee and Plant Diversity

Overall, 122 wild bee species were identified among the 1491 individuals collected during this study (Table S3). Sampling completeness, assessed using the Chao 1 estimator, was 71.9% for Baratz, 87.4% for Ottava, 73.4% for Cala d'Oliva, and 76.6% for Fornelli, and rarefaction curves indicate that some species were missed despite the extensive sampling (Figure S3). Bee richness peaked at all sites in May–July except in Cala d'Oliva where richness was almost identical in February–April and May–July, while the difference was not significant in Fornelli between these periods (Table 1, Table S4). Richness decreased strongly in August–November at Baratz and Ottava, while essentially no bees (since almost no flowering plants) were observed in such periods at the Asinara sites.

**TABLE 1:** Observed abundance (*N* = number of observations for the networks, average number of individuals per plot for total plants in the communities), species richness (*S*), Shannon–Weaver diversity (*H′*) and Gini‐Simpson dominance (*GS*) of the studied networks and communities, divided by site and period.

	Period	*N*	*S*	*H*	*GS*
*Bees*					
Baratz	Feb‐Apr	110	28	2.85	0.91
	May‐Jul	112	43	3.52	0.96
	Aug‐Nov	47	13	2.30	0.86
Ottava	Feb‐Apr	276	30	2.66	0.90
	May‐Jul	362	72	3.43	0.92
	Aug‐Nov	35	15	2.58	0.90
Cala d'Oliva	Feb‐Apr	119	32	3.03	0.92
	May‐Jul	192	31	2.43	0.80
Fornelli	Feb‐Apr	74	20	2.74	0.91
	May‐Jul	164	30	2.75	0.88
*Plants (network)*					
Baratz	Feb‐Apr	110	16	1.89	0.73
	May‐Jul	112	20	2.61	0.91
	Aug‐Nov	47	6	1.47	0.73
Ottava	Feb‐Apr	276	26	2.35	0.87
	May‐Jul	362	33	2.83	0.91
	Aug‐Nov	35	10	1.93	0.81
Cala d'Oliva	Feb‐Apr	119	23	2.56	0.89
	May‐Jul	192	13	1.65	0.66
Fornelli	Feb‐Apr	74	11	1.84	0.79
	May‐Jul	164	12	1.98	0.82
*Plants (community)*					
Baratz	Feb‐Apr	25.58	92	3.76	0.97
	May‐Jul	27.00	89	3.64	0.96
	Aug‐Nov	15.72	24	2.01	0.74
Ottava	Feb‐Apr	536.96	69	3.43	0.95
	May‐Jul	385.81	86	3.62	0.96
	Aug‐Nov	35.24	33	2.54	0.89
Cala d'Oliva	Feb‐Apr	1239.08	69	3.50	0.96
	May‐Jul	129.89	45	2.67	0.89
Fornelli	Feb‐Apr	1091.97	75	3.64	0.97
	May‐Jul	173.56	40	2.26	0.84

*Note:* The *N* value for total flower cover was calculated as the sum of the % cover of each plant species across plots, years, and periods.

The collected specimens belonged to 27 genera and 6 families (Table S3). Apidae was the most abundant family (624 individuals), whereas Melittidae was the least represented (2 individuals). *Bombus terrestris* was the most abundant species at both sites in Asinara island (176 individuals), whereas *Ceratina cucurbitina* was the most abundant species at both Nurra sites (150 individuals). Notably, *Hoplitis corsaria* (Warncke, 1991) was recorded for the first time in Italy, representing a significant addition to the national bee fauna (Table S3). In addition, six species ( *Andrena dorsata* , *Lasioglossum marginatum* , *Lasioglossum medinai*, *Osmia leaiana* , *Osmia scutellaris* , and *Nomada flavoguttata* ) were newly recorded for the island of Sardinia (Table S3). Details of the collection data for these seven species are provided in Table S5.

A total of 196 plant species (whole community) were recorded overall at the four sampling sites (Table S6). The % of these species visited by bees ranged from 14.7% at Fornelli in February–April to 38.4% at Ottava in May–July. In general, between 20% and 30% of all plants were used on average across sites (averages across periods). Species richness of plants used by bees varied across periods at four sites, especially decreasing from spring–early summer to late summer at the Nurra sites, though not at the Asinara sites (Table 1, Table S4). A lack of a similar trend also appeared for the richness of all plant species (Table 1, Table S4). However, the % of used plants by bees seemed not to follow a recognizable temporal trend (e.g., peak of % plant used by bees being highest in February–April at Cala d'Oliva but in August–November at Baratz) (Table 1).

The Shannon–Wiener and Gini–Simpson indices varied across the monitored sites and periods for both wild bees and visited plants similarly to what was observed for species richness, with higher values of diversity and dominance in May–July at the sites located in Nurra (Ottava and Baratz), while similar values between February–April and May–July were observed at the sites in Asinara (Cala d'Oliva and Fornelli) (Table 1). Similar trends were observed for the whole plant communities.

### 3.3 Bee–Plant Networks

In total, we recorded 1491 interactions between bees and flowering plants, distributed as follows: Ottava 673, Cala d'Oliva 311, Baratz 269, and Fornelli 238. Networks showed some shared characteristics among sites and periods, as well as some differences (see an example for the three periods of Baratz; Figures 2, 3, 4, Figures S4–S10 for all the other networks).

**FIGURE 2:**
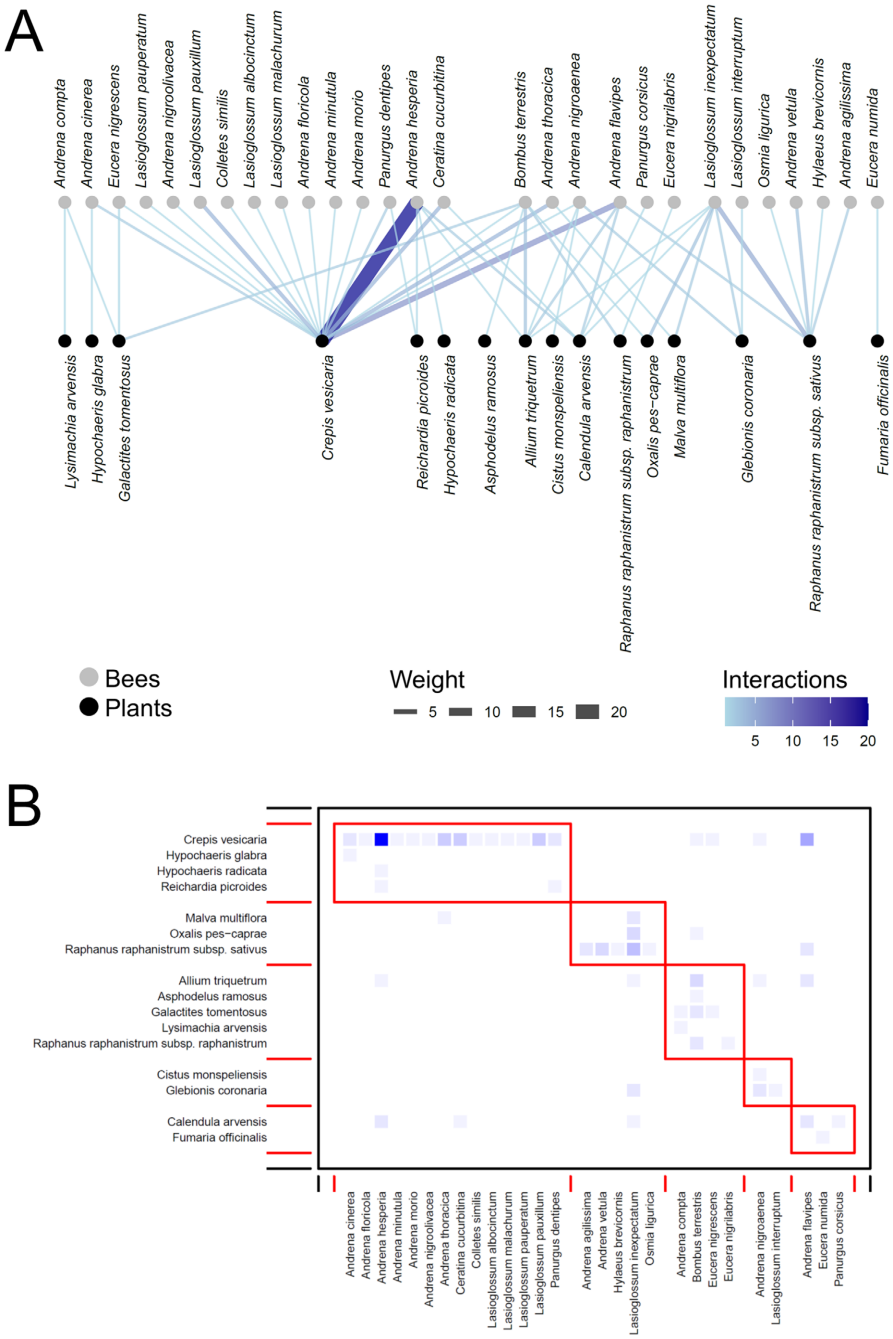
(A) Bipartite network graph depicting the network analysis for the bee‐plant network at Baratz in February–April. Link width and blue shading level indicates the frequency of visits to plant species by a given bee species. (B) Modularity of the bee‐plant network at Baratz in February–April. The *y*‐axis represents the plants and the *x*‐axis represents the bees. The red rectangles are the communities that emerged through modularity. The frequencies of interactions—where they differ from zero—between each bee species and each plant species increase from light blue to dark blue.

**FIGURE 3:**
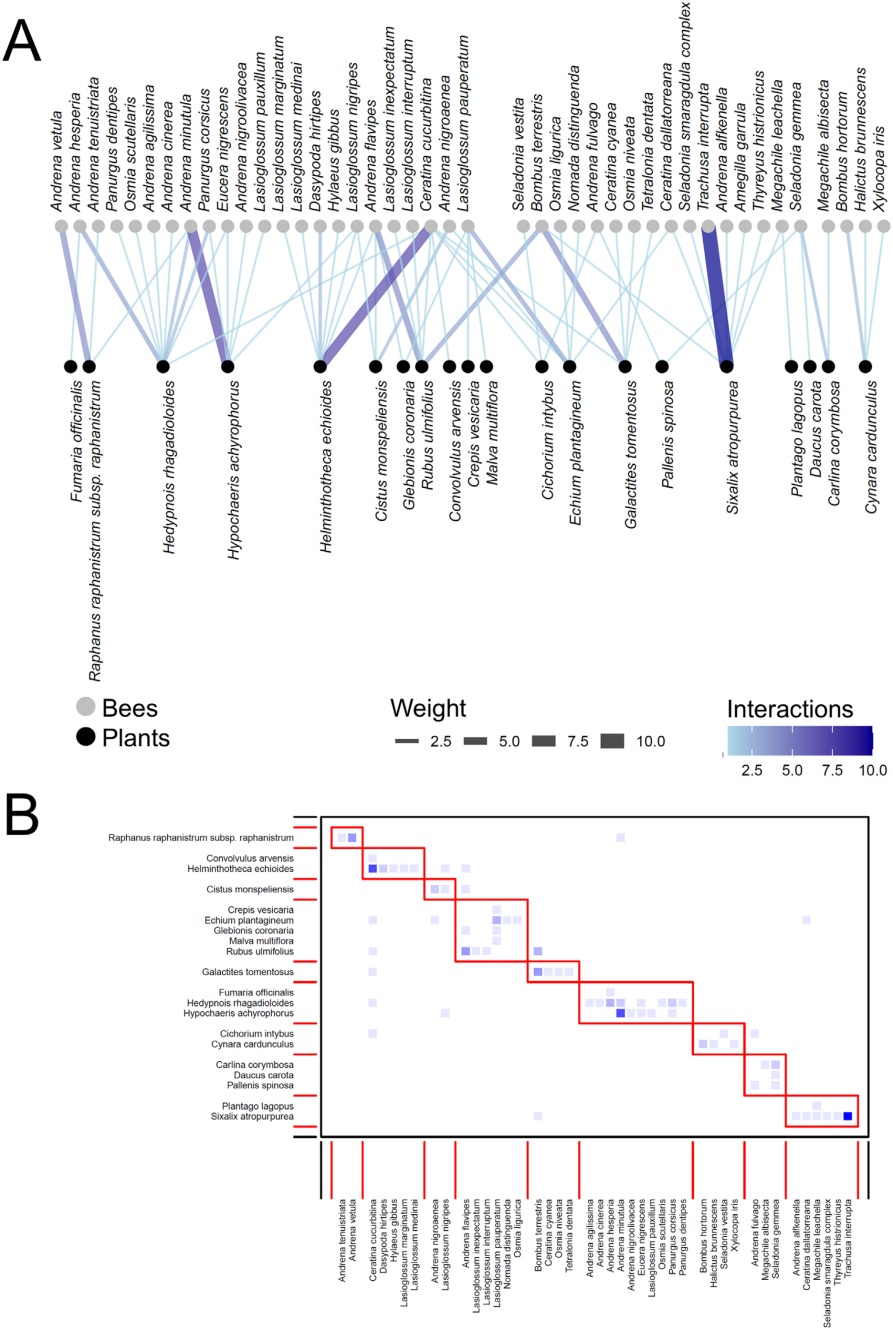
(A) Bipartite network graph depicting the network analysis for the bee‐plant network at Baratz in May–July. Link width and blue shading level indicates the frequency of visits to plant species by a given bee species. (B) Modularity of the bee‐plant network at Baratz in May–July. The *y*‐axis represents the plants and the *x*‐axis represents the bees. The red rectangles are the communities that emerged through modularity. The frequencies of interactions—where they differ from zero—between each bee species and each plant species increase from light blue to dark blue.

**FIGURE 4:**
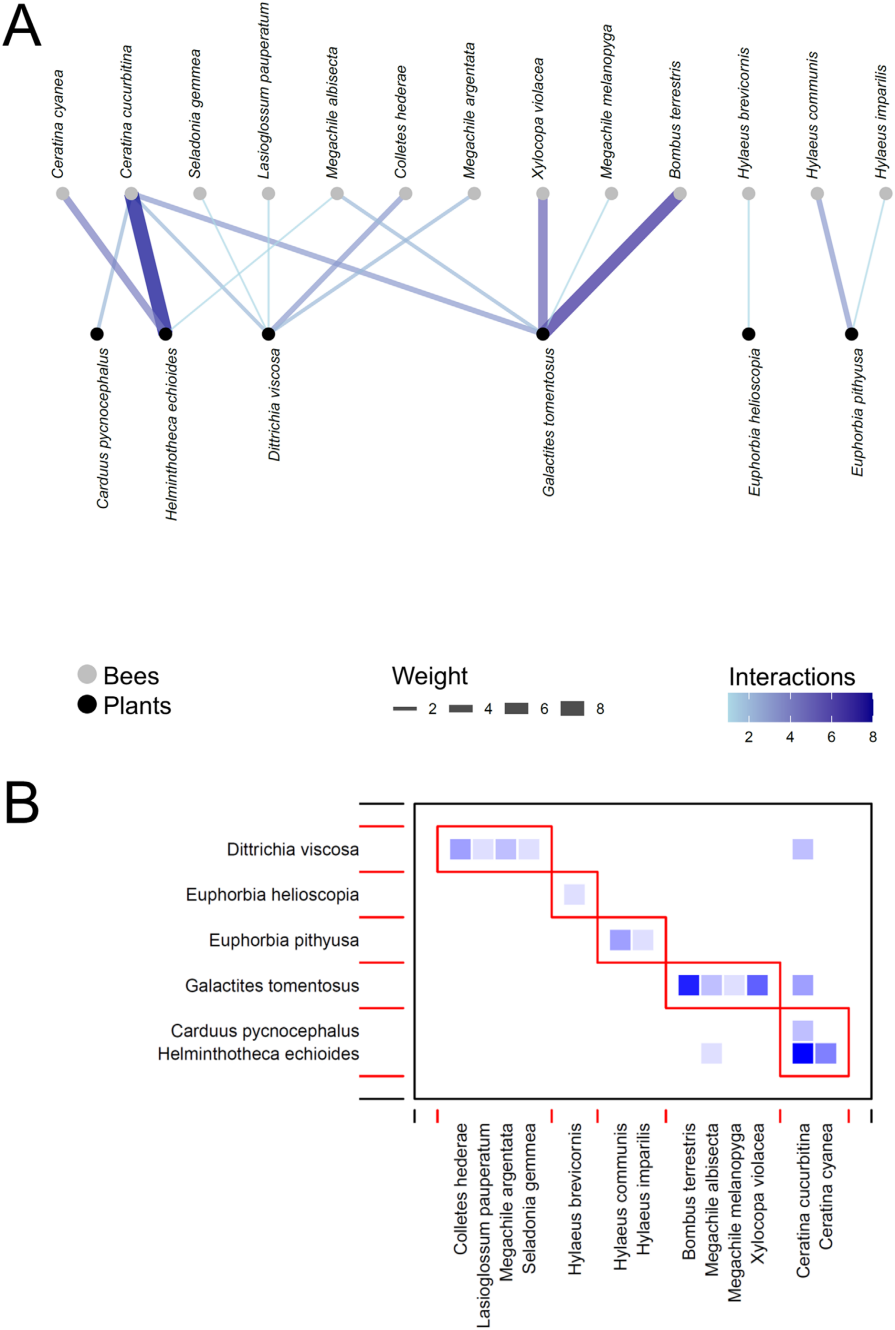
(A) Bipartite network graph depicting the network analysis for the bee‐plant network at Baratz in August–November. Link width and blue shading level indicates the frequency of visits to plant species by a given bee species. (B) Modularity of the bee‐plant network at Baratz in August–November. The *y*‐axis represents the plants and the *x*‐axis represents the bees. The red rectangles are the communities that emerged through modularity. The frequencies of interactions—where they differ from zero—between each bee species and each plant species increase from light blue to dark blue.

The network–level indices of specialization (*H*_*2*_ *′*), weighted nestedness (*WNODF*) and modularity (*M*) showed significant deviations from random expectations at essentially all sites and periods (Table 2). However, while the observed *H*_*2*_ *′* and *M* values were higher than those of their respective null models, *WNODF* values were consistently lower (Table 2). Thus, the networks were highly specialized, modular but not nested.

**TABLE 2:** Values of the network‐level indices of specialization (*H*_*2*_ *′*), nestedness (*WNODF*), and modularity Q (*M*), together with their comparisons with random networks based on standardized effect sizes (*z‐*scores) obtained from null models.

Index	Site	Period	Observed	*z‐*score	*p*
*H2′*	Baratz	Feb‐Apr	0.39	6.32	**< 0.001**
		May‐Jul	0.55	17.81	**< 0.001**
		Aug‐Nov	0.66	14.19	**< 0.001**
	Ottava	Feb‐Apr	0.43	30.42	**< 0.001**
		May‐Jul	0.44	18.38	**< 0.001**
		Aug‐Nov	0.46	2.95	**< 0.001**
	Cala d'Oliva	Feb‐Apr	0.40	11.18	**< 0.001**
		May‐Jul	0.56	22.10	**< 0.001**
	Fornelli	Feb‐Apr	0.51	6.19	**< 0.001**
		May‐Jul	0.62	24.46	**< 0.001**
*WNODF*	Baratz	Feb‐Apr	16.80	−0.78	0.60
		May‐Jul	3.61	−2.83	**< 0.001**
		Aug‐Nov	8.96	−3.30	**< 0.001**
	Ottava	Feb‐Apr	14.80	−6.12	**< 0.001**
		May‐Jul	5.38	−5.83	**< 0.001**
		Aug‐Nov	10.00	0.17	1.00
	Cala d'Oliva	Feb‐Apr	7.11	−2.38	**< 0.001**
		May‐Jul	13.79	−3.80	**< 0.001**
	Fornelli	Feb‐Apr	11.13	−2.04	**< 0.001**
		May‐Jul	9.17	−5.08	**< 0.001**
*M*	Baratz	Feb‐Apr	0.44	8.86	**< 0.001**
		May‐Jul	0.70	6.14	**< 0.001**
		Aug‐Nov	0.58	9.93	**< 0.001**
	Ottava	Feb‐Apr	0.48	16.93	**< 0.001**
		May‐Jul	0.53	25.67	**< 0.001**
		Aug‐Nov	0.61	6.74	**< 0.001**
	Cala d'Oliva	Feb‐Apr	0.55	9.82	**< 0.001**
		May‐Jul	0.48	18.57	**< 0.001**
	Fornelli	Feb‐Apr	0.51	9.13	**< 0.001**
		May‐Jul	0.60	16.78	**< 0.001**

*Note:* In bold, the significant comparisons.

The *z*–values inspections revealed differences between sites and periods within sites (Table 2, Figure S11). Indeed, it was apparent how in most sites networks reached the highest specialization and modularity in May–July, decreasing in complexity and number of interactions in August–November (Figures 2, 3, 4, Figures S4–S10). Such differences seemed to be more accentuated in Baratz and Ottava compared with the two Asinara sites, where February–April networks were similar to those of May–July (Figure S11). The number of modules in the networks seemed to peak at May–July in most sites (Figures 2, 3, 4, Figures S4–S10). Across sites, February–April had the most specialized networks in Ottava, while May–July had the most specialized networks in the Asinara sites, with similar trends also found for modularity (Figure S11). Values showing lack of nestedness were very variable across sites and periods, though in general (except in Ottava) decreasing from spring forward (Figure S11).

**FIGURE 5:**
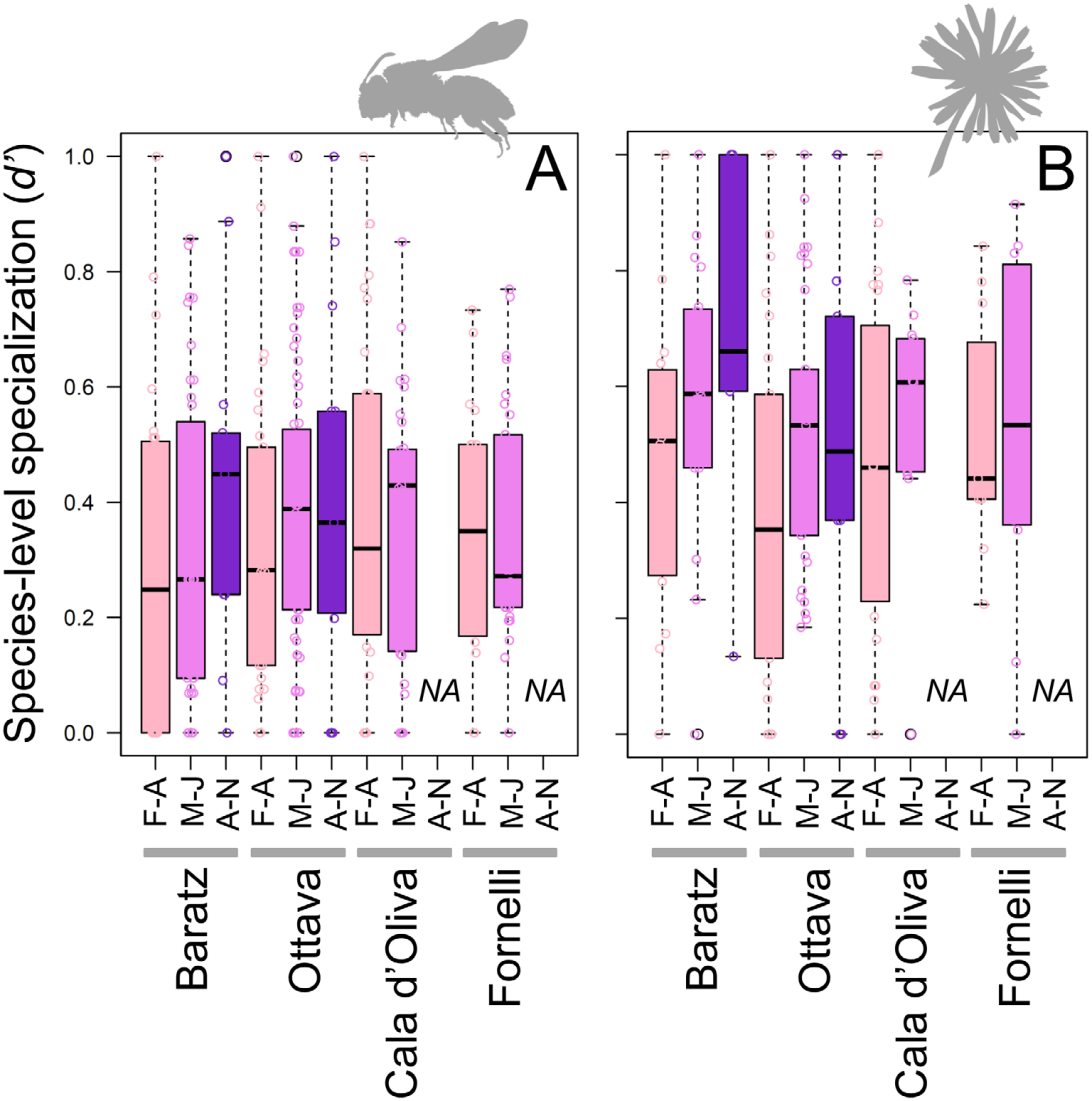
Box and Whisker plots showing the minimum‐maximum, first‐third quartiles, and median of both bee (A) and plant (B) species‐level specialization (*d'*) for the all bee‐plant networks. F‐A: February–April, M‐J: May–July, A‐N: August–November. Networks lack in August–November at both Asinara sites, and in the plots, NA are shown in these cases. Bee and plant silhouettes were retrieved from https://www.phylopic.org, under Public Domain.

The level of specialization of each bee and plant species was assessed using the *d'* index (Supporting Information S1—DATASET_SpeciesLevel.xlsx). In all plant–pollinator networks, the *d'* specialization index values were very variables, with median values between 0.2 and 0.5 for bees (Figure 5A) and between 0.3 and 0.7 for plants (Figure 5B). No apparent temporal trends appeared in *d'* for bees (Figure 5A), with values either lower in spring (e.g., Ottava) or in summer (e.g., Fornelli), while perhaps a certain trend of increasing *d'* in summer can be recognized for plants (Figure 5B). However, variability was extremely high within networks, so that no significant effects of period or site emerged (see below). A greater number of *d'* > 0.8 and a lower number of *d'* < 0.2 for plants compared with bees seemed to suggest that plants were overall slightly more specialized than bees in the networks (Figure 5A,B). The rare occurrence of both highly generalists and extremely specialized species also agrees with the general lack of nested patterns in the studied networks.

### 3.4 Potential Environmental Effects on Variation in Community and Network Indices

**FIGURE 6:**
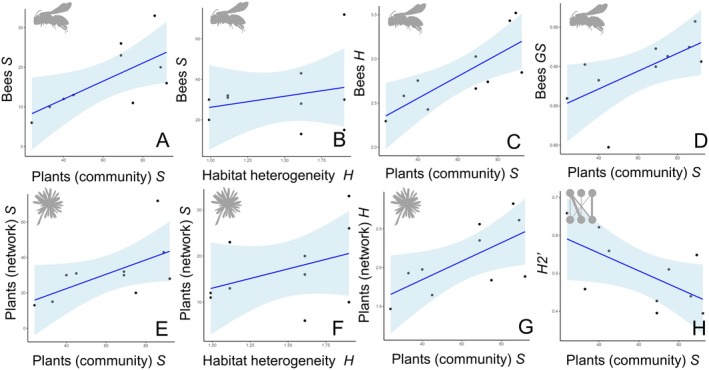
Scatter plots showing the relationships between (A) whole plant community richness and bee richness, (B) habitat heterogeneity and bee richness, (C) whole plant community richness and bee diversity, (D) whole plant community richness and bee dominance, (E) whole plant community richness and visited plant richness, (F) habitat heterogeneity and visited plant richness, (G) whole plant community richness and visited plant diversity, and (H) whole plant community richness and network specialization. Lines represent adjustments to linear models, while blue interval depict 95% confidence limits. Bee and plant silhouettes were retrieved from https://www.phylopic.org, under Public Domain.

Some of the found differences in community–level indices among sites and periods may be potentially explained by variation in plant communities and habitats. Indeed, the simple linear regressions showed a positive relationship between plant community richness and bee richness, bee diversity and bee dominance (Figure 6A,C,D, Table 3). Bee richness was also positively associated with habitat heterogeneity (Figure 6B, Table 3). Plant community richness and habitat heterogeneity showed a positive association with richness of plant species visited by bees (Figure 6E,F, Table 3), and only plant community richness was positively related with diversity of plant species visited by bees (Figure 6G, Table 3). Furthermore, a marginally significant effect of plant community richness on network–level specialization was observed (*p* = 0.056) (Figure 6H, Table 3). Species–level specialization index in the network (*d'*), likely due to their high within–network variability (see above), on the other hand, did not depend on any traits of the plant community or habitat, and no effect of site and period or their interaction appeared (Table 3).

**TABLE 3:** Models.

Index	Model and factors	Estimate	SE	*t* or *z*	*p*
*Network‐ and community‐level*					
*S* (bees)	Plants (community) *N*	−0.002	0.009	−0.173	0.862
	Plants (community) *S*	0.014	0.003	5.317	**< 0.001**
	Habitat heterogeneity *H*	0.352	0.157	2.236	**0.025**
*H* (bees)	Plants (community) *N*	0.005	0.022	0.231	0.823
	Plants (community) *S*	0.012	0.004	3.44	**0.009**
	Habitat heterogeneity *H*	0.194	0.360	0.538	0.605
*GS* (bees)	Plants (community) *N*	0.001	0.002	0.613	0.557
	Plants (community) *S*	0.001	0.0005	2.393	**0.044**
	Habitat heterogeneity *H*	0.034	0.038	0.899	0.395
*S* (plants)	Plants (community) *N*	0.018	0.012	1.489	0.136
	Plants (community) *S*	0.014	0.004	4.056	**< 0.001**
	Habitat heterogeneity *H*	0.502	0.216	2.319	**0.020**
*H* (plants)	Plants (community) *N*	0.019	0.024	0.793	0.451
	Plants (community) *S*	0.012	0.005	2.448	**0.040**
	Habitat heterogeneity *H*	0.363	0.395	0.921	0.384
*GS* (plants)	Plants (community) *N*	0.004	0.005	0.935	0.377
	Plants (community) *S*	0.002	0.001	1.439	0.188
	Habitat heterogeneity *H*	0.065	0.075	0.876	0.406
*H2′*	Plants (community) *N*	−0.005	0.005	−1.099	0.304
	Plants (community) *S*	−0.002	0.001	−2.234	0.056
	Habitat heterogeneity *H*	−0.081	0.081	−1.004	0.345
*WNODF*	Plants (community) *N*	0.047	0.237	−0.198	0.847
	Plants (community) *S*	−0.011	0.059	−0.177	0.864
	Habitat heterogeneity *H*	−0.357	3.840	−0.093	0.928
*M*	Plants (community) *N*	−0.003	0.004	−0.664	0.526
	Plants (community) *S*	−0.0005	0.001	0.500	0.631
	Habitat heterogeneity *H*	0.014	0.068	0.205	0.843
*Species‐level*					
*d'* (bees)	Plants (community) *S*	0.009	0.015	0.616	0.538
	Plants (community) *N*	0.053	0.073	0.726	0.468
	Habitat heterogeneity *H*	−0.474	0.818	−0.579	0.563
		Deviance	Resid. Dev	*F*	*p*
	Period	0.183	19.665	1.418	0.243
	Site	0.009	19.655	0.073	0.929
	Period × Site	0.026	19.628	0.209	0.811
*d'* (plants)	Plants (community) *S*	0.015	0.021	0.713	0.477
	Plants (community) *N*	0.084	0.106	0.800	0.425
	Habitat heterogeneity *H*	−1.245	1.159	−1.074	0.284
		Deviance	Resid. Dev	*F*	*p*
	Period	0.354	11.152	2.570	0.079
	Site	0.44	11.107	0.320	0.726
	Period × Site	0.075	11.032	0.547	0.579

*Note:* *t*‐value is expressed for Gaussian models (continuous data), while *z*‐value is expressed for Poisson models (for richness, *S*). The network‐ and community‐level models are individual linear regressions (each explanatory variable is treated separately), while the species‐level models are generalized linear models (GLMs) with all listed factors included. For the species‐level models, the overall effects of period, site and their interactions were shown following an ANOVA performed on the model, since no paired differences between sites, periods or interactions were detected in the GLMs.

## 4 Discussion

In this study, we carried out a 3‐year monitoring activity to examine wild bee–plant interaction patterns across two distinct areas of Sardinia (the main island and a small satellite island). Sardinia is overall the second largest Mediterranean island but one of the less studied areas in Southern Europe concerning bee diversity (e.g., Alfken 1938; Nobile et al. 2021; Annessi et al. 2025) and for which no studies to date investigated bee–plant networks. We established an essential baseline data for the island and performed evaluations of potential variations in wild bee diversity and bee–plant interactions across islands, habitats and land–use types.

### 4.1 Bee and Plant Diversity

Our bee sampling effort recorded a total of 122 species. Notably, *Hoplitis corsaria* (Warncke, 1991), previously reported only from Corsica (Le Divelec et al. 2024), is here recorded for the first time from Italy. In addition, six further species represent new records for Sardinia (Table S5) (Nobile et al. 2021; Reverté et al. 2023; Cornalba et al. 2024), highlighting the still partially unexplored bee fauna of the island. Confirming this, a recent survey conducted in a northeastern area of Sardinia (Coluccia Peninsula) by Annessi et al. (2025) reported six additional wild bee species as new records for the island. Notably, despite the strong sampling effort, it seems that around 20% (average across sites) of bee species likely occurring in the studied area were not collected, highlighting how diverse bee communities can be in Sardinia.

The comparison of the four monitored sites across the sampled periods—though preliminarily—suggests interesting relationships between the diversity of both plants and bees and environmental traits. In particular, bee and plant species richness seemed to be higher where habitat heterogeneity and overall richness of plants in the community are also higher. In general, the greater the diversity of plants (including allodiversity due to the presence of non–native cultivated plants or even weeds of crops), the greater the diversity of bees is (e.g., Gerner and Sargent 2022; Philpott et al. 2025), given their role as potential pollinators, so that similar trends for bees and plants could be expected. Shannon diversity also followed the same trend, while dominance followed this trend for bees only. On the other hand, we did not found any effect of flower cover, differently to what observed in previous studies (e.g., Hyjazie and Sargent 2022) Additionally, habitat heterogeneity was previously shown to positively affect bee and plant diversity (e.g., Parreño et al. 2024; Zanini et al. 2024). It is important to note that high species diversity promotes not only the resilience (i.e., the ability to return quickly to equilibrium after disturbances) of mutualistic communities but also their persistence (i.e., the proportion of coexisting species to the total number of species once equilibrium is reached) (Thébault and Fontaine 2010).

The greater habitat and land–uses diversity observed at Ottava and Baratz is undoubtedly due to anthropogenic activities, including suburban developments and low–intensity agricultural practices such as grazed grasslands, small orchards and gardens. In general, human disturbance can significantly reduce the abundance and species richness of wild, unmanaged bees, but this is true mainly in systems that, due to human activity, experience extreme habitat loss (Winfree et al. 2009; Stein et al. 2018, 2020; Maurer et al. 2024). Hence, landscape diversity and moderate anthropogenic land uses may be compatible not only with the conservation of many bee species but can also support a more abundant and diverse wild bee community, as already observed in forest ecosystems (Winfree et al. 2007). This is likely because many crops and constructed–urban habitats can provide many diverse floral resources and potentially offer an overall longer flowering period. This finding is further supported by the greater richness and diversity of pollen collected by honeybees in landscapes covered by more diverse habitat (Montoya‐Pfeiffer et al. 2021; Satta et al. 2024).

The observed differences between sites can also depend on the differential phenology of overall flowering plants between main Sardinia and Asinara, with the former still having a number of plants in bloom in November while the latter almost not having any plant in bloom in autumn. Accordingly, bees are much more active—and their diversity was higher—at Baratz and Ottava compared with Cala d'Oliva and Fornelli in May–July, since summer seemed to represent the last bee flight period for the latter sites. Additionally, the observed patterns can, perhaps, also arise from the large difference in size between the two islands, with the main Sardinia sites having indeed more species and higher diversity than the much smaller Asinara (Traveset et al. 2016; Wang et al. 2020). Further studies focused on a greater number of locations are needed in order to disentangle island and habitat–effects.

Most of the species we collected in high abundance in all monitored sites belonged to the Andrenidae family (Table S3). At the lower end of the abundance scale, 26 of the bee species collected were single occurrences, and these were evenly distributed among the five well‐represented families (Table S3). This indicates that the wild bee community in north‐western Sardinia includes a relatively high number of rare species, and that long‐term sampling is required to obtain an accurate picture of the local diversity. Anyway, none of the species we collected both in high and low numbers were listed in the Italian Red List of bees (Quaranta et al. 2018).

According to the hypothesis that social species are more sensitive to human disturbance than solitary species (Winfree et al. 2009), we found that *Bombus terrestris* (a social apid species) was the most abundant species in the Asinara sites (Cala d'Oliva and Fornelli), whereas *Ceratina cucurbitina* (a solitary apid species) was the most abundant species in the Nurra sites (Baratz and Ottava). However, disturbance effects on social vs solitary bee species is still not fully clear. While Winfree et al. (2009) observed a negative effect on social, but not on solitary bees, differences between the two groups were not significant. Furthermore, highly urbanized contexts (i.e., with greater green area fragmentation), seem to harbor higher proportion of both social species and large–bodied bees, compared with solitary, small species (reviewed in Ferrari and Polidori 2022). It is likely that these different trends depend at least partially by the type of land–use disturbance and other still poorly explored factors, such as microclimatic conditions or abundance of nesting resources, so that further studies are needed to better understand how human–driven habitat modifications alter the communities of wild bees.

### 4.2 Plant–Pollinator Interactions

In all sites and periods, the plant–pollinator networks studied exhibited a strong reliance on specific interactions (high specialization, or complementarity), meaning that plants tend to interact with a limited number of pollinators and *vice versa*. This suggests a high degree of co–adaptation or coevolution between specific species, possibly driven by local ecological pressures or highly specialized environments. Mediterranean environments are known for their unique climatic conditions (i.e., hot, dry summers and mild, wet winters) involving for most plants a flowering period concentrated between late winter and early spring (Bosch et al. 1997). In these environments, many plant and pollinator species, may have evolved highly specialized interactions to maximize resource acquisition in a competitive and seasonally harsh environment.

Specialization may also have been driven by the high biodiversity typical of Mediterranean regions (Michener 1979; Nieto et al. 2014; Winfree 2010; Bartomeus et al. 2018), where species may develop more exclusive interactions to reduce competition and enhance ecological niches. Network specialization was, however, variable across periods, peaking in summer (Asinara) to autumn (main Sardinia) at all sites, while being essentially similar across sites at any period. Since summer and autumn (respectively for Asinara and main Sardinia) were also the late activity periods for bees and flowering periods for plants, this pattern may suggest that poorer communities in terms of richness favor greater complementarity in resource use. Ecological network specialization was previously seen to increase when the number of species decreases (Schleuning et al. 2012), particularly in the context of habitat loss and fragmentation (Zhang et al. 2023). Accordingly, we have found a marginal decrease of network specialization with whole–community plant species richness.

Another distinctive feature of these networks is that they were not nested. Plant–pollinator networks are often described as nested, meaning that specialists interact with subsets of the partners that generalists interact with, forming a hierarchical structure (Bascompte et al. 2003). Nestedness has always been believed to be the most important determinant of mutualistic network stability (Memmott et al. 2004). The absence of this pattern in the Mediterranean networks was also reported by Ropars et al. (2020) and for *Bombus*–plant networks in the only study carried out to date in Sicily, the largest Mediterranean island (Polidori et al. 2025). Disturbance regimes typical of Mediterranean environments, such as fires, droughts, and grazing, could disrupt and prevent the formation of nested structures by forcing species to adapt to localized conditions and limiting the potential for widespread generalist interactions. However, generalist species that can tolerate human–altered landscapes and human activity tend to thrive in urban habitats (Prendergast et al. 2022). This fragmentation of interactions may also reflect the varying phenological schedules of plants and pollinators in such environments, where species are active during different times of the year, further limiting the possibility of nested interactions. Furthermore, it was shown that higher levels of nestedness are associated with a higher temperature seasonality (Song et al. 2017), because nested structures of plant–pollinator networks could be more advantageous under highly seasonal environments. The Mediterranean basin, especially on the coasts and the islands, has typically a less seasonal amplitude (warm to hot summers and mild winters), compared with inland and northern areas (warm to hot summers and cold winters) (Joffre and Rambal 2001), thus possibly agreeing with the lack of nested patterns found in the studied Sardinian sites.

The high modularity observed in all our studied networks was another significant structural feature. Modular networks are characterized by groups of species (modules) that interact more frequently with each other than with species outside their module (Olesen et al. 2007). Little is known about the implications of the modular structure on network stability. Thébault and Fontaine (2010) have emphasized that the modular pattern has a negative effect on the persistence and resilience of mutualistic networks, whereas Grilli et al. (2016) reported that modularity is often linked to functional and ecological stability. It is difficult to suggest the cause of the observed variation in the modularity of the networks, generally most pronounced in May–July but not differing among sites, since modularity was not significantly associated with any environmental variable in our models. The high modularity detected in our studied networks is in weak accordance with the hypothesis of Olesen et al. (2007), which suggests that such pattern arises when the numbers of plant and pollinator species are high (> 150), whereas in their analysis the networks with < 50 species were never modular. Our modularity values (ranging from 0.44 to 0.70) were comparable to those reported by Olesen et al. (2007) (average = 0.51, *n* = 29 significantly modular networks), despite our species richness (bees + plants) ranging from 19 to 105. The number of modules and the level of modularity observed in pollination networks are found to be invariant to sampling effort at different times (Dupont and Olesen 2012).

Altogether, the values of the analyzed network–level metrics give us insights on the stability of the observed networks. Following Thébault and Fontaine (2010), in general, a stable bipartite mutualistic network is specialized (which is the case in our sites) and has a nested but not highly modular topology (which is the opposite trend compared with our sites). Hence, the studied Sardinian networks, although rich in species, may not be very stable and somehow vulnerable. A similar general pattern was found in networks recently studied by Gay et al. (2024) in meadows within French intensive agricultural landscapes that appeared to be resilient thanks to the high number of species they host (Okuyama and Holland 2008), but also possibly having a certain vulnerability because of the low amount of shared partners. Interestingly, the small island of Asinara and the large main Sardinia essentially show very weak differences in network topologies, while it is expected that smaller islands harbor simplified networks with great overlap among nodes (Traveset et al. 2016). Perhaps, the different levels of anthropogenic pressure on the two areas are masking a potential island size effect, but new studies are necessary to test for such a hypothesis. In addition to island size and anthropogenic pressure, another factor that may potentially influence network metrics is the presence of managed honey bees ( *Apis mellifera* ), which were excluded from our analyses (Pasquali et al. 2025). Honey bees can strongly affect plant–pollinator networks, particularly under high colony densities, by increasing interaction redundancy, altering patterns of resource use, or reshaping species roles within the network (Valido et al. 2019). In our study system, however, honey bees were absent from Asinara Island and occurred at low densities in the mainland sites. Under such conditions, their influence on network structure is likely limited.

Concerning the species–level network indices, we evidenced a variable value of specialization (*d'*), with relatively few species having high or low specialization and most having intermediate values. This agrees with the lack of nestedness of the networks, since in nested motifs highly specialist species often interact with some of the most generalized species (Bascompte et al. 2003). Such great variability likely explains why no effects of vegetation or habitat variables, as well as of site and period, emerged from our models. Longer flowering periods were previously negatively related to bees' *d*', as the longer the flowers are available for bee visits, the higher is their probability to be visited by different species (Lázaro et al. 2020). Plant species were on average more specialized than bee species, as previously shown in other networks but not universally (Blüthgen et al. 2006).

## 5 Conclusions

The studied communities revealed a great diversity of bee species in an overlook, albeit large, Mediterranean island, with such diversity perhaps being at least partially driven by the presence of specific habitats, land–use types, and flowering species availability. In parallel, despite sharing common structural traits, the ecological networks studied in Sardinia exhibited some differences that we may also preliminarily suggest to be shaped, at least partially, by the presence of flowering species availability, though apparently not by land use types. Further studies encompassing a larger number of locations across Sardinia are needed to more precisely test for habitat–driven effects on both community structures and network topologies.

## Author Contributions

**Matteo Lezzeri:** conceptualization (equal), data curation (equal), investigation (equal), writing – original draft (equal). **Vanessa Lozano:** conceptualization (equal), data curation (equal), investigation (equal), writing – original draft (equal). **Giuseppe Brundu:** conceptualization (equal), funding acquisition (equal), project administration (equal), writing – original draft (equal). **Simone Flaminio:** data curation (equal), investigation (equal). **Ignazio Floris:** conceptualization (equal), project administration (equal), supervision (equal), writing – review and editing (equal). **Stephane Knoll:** data curation (equal), investigation (equal). **Michelina Pusceddu:** conceptualization (equal), data curation (equal), investigation (equal), project administration (equal), writing – review and editing (equal). **Marino Quaranta:** conceptualization (equal), data curation (equal), investigation (equal), writing – review and editing (equal). **Carlo Polidori:** conceptualization (equal), data curation (equal), formal analysis (equal), writing – original draft (equal). **Alberto Satta:** conceptualization (equal), funding acquisition (equal), project administration (equal), resources (equal), writing – original draft (equal).

## Funding

Study financed, in part, with funds from the Asinara National Park—Directive of the Italian Ministry of Environment for activities directed to the conservation of biodiversity—Directive 2020—Activities directed to the conservation of pollinators—Pollinator insects in the Asinara National Park. Asinara National Park—Directive of the Italian Ministry of Environment for activities directed to the conservation of biodiversity—Directive 2020. Study partially funded under BeeNet project (2019–2023), funded through the FEASR 2014–2020 program (European Agricultural Fund for Rural Development), and overseen by RRN (Rete Rurale Nazionale) and MASAF (Ministry of Agriculture, Food Sovereignty, and Forestry). This study was also partially funded under the National Recovery and Resilience Plan (NRRP), Mission 4 Component 2 Investment 1.4—Call for tender No. 3138 of 16 December 2021, rectified by Decree n. 3175 of 18 December 2021 of the Italian Ministry of University and Research funded by the European Union—NextGenerationEU; Award Number: Project code CN_00000033, Concession Decree No. 1034 of 17 June 2022 adopted by the Italian Ministry of University and Research, CUP J83C22000870007, Project title “National Biodiversity Future Center—NBFC”.

## Conflicts of Interest

The authors declare no conflicts of interest.

## Supporting information


**Table S1:** Location and elevation of the four sampling sites (north‐western Sardinia, Italy).
**Table S2:** Habitat composition (%) of the four sampling sites (north‐western Sardinia, Italy).
**Table S3:** List of the wild bee species collected by standardized transect walks during 3 years of sampling (2021‐2022‐2023) across the seasons (February to November) and in the four sites of the study area (Northwest Sardinia, Italy).
**Table S4:** Differences in the observed species richness (*S*), Shannon–Wiener diversity (*H*) and Gini‐Simpson dominance (*GS*) of the studied communities across periods.
**Table S5:** Collection data on wild bee species identified as new records for Sardinia and, in the case of *Hoplitis corsaria* (Warncke, 1991), also for Italy.
**Table S6:** List of plant species at the four sampling sites. The gray color of the cell indicates the presence of the plant species at that site, while the “×” signifies that the species has been visited by at least one wild bee.
**Figure S1:** Temporal patterns of whole community plant diversity (as maximum number of species recorded *per* plot across 3 years‐period) from February (F) to November (N), at each of the four studied sites.
**Figure S2:** Correlation matrix showing the linear relationships between all the vegetation‐associated and habitat‐associated variables considered in this study. The scale bar represents the Pearson correlation coefficient. Only the significant correlations (*p* < 0.05) are shown.
**Figure S3:** Rarefaction and extrapolation curves for species diversity (richness) of bee species in the four sites. Shaded areas are 95% confidence intervals.
**Figure S4:** A, Bipartite network graph depicting the network analysis for the bee‐plant network at Ottava in February–April. Link width and blue shading level indicates the frequency of visits to plant species by a given bee species. B, Modularity of the bee‐plant network at Ottava in February–April. The *y*‐axis represents the bees and the *x*‐axis represents the plants. The red rectangles are the communities that emerged through modularity. The frequencies of interactions—where they differ from zero—between each bee species and each plant species increase from light blue to dark blue.
**Figure S5:** A, Bipartite network graph depicting the network analysis for the bee‐plant network at Ottava in May–July. Link width and blue shading level indicates the frequency of visits to plant species by a given bee species. B, Modularity of the bee‐plant network at Ottava in May–July. The *y*‐axis represents the plants and the *x*‐axis represents the bees. The red rectangles are the communities that emerged through modularity. The frequencies of interactions—where they differ from zero—between each bee species and each plant species increase from light blue to dark blue.
**Figure S6:** A, Bipartite network graph depicting the network analysis for the bee‐plant network at Ottava in August–November. Link width and blue shading level indicates the frequency of visits to plant species by a given bee species. B, Modularity of the bee‐plant network at Ottava in August–November. The *y*‐axis represents the plants and the *x*‐axis represents the bees. The red rectangles are the communities that emerged through modularity. The frequencies of interactions—where they differ from zero—between each bee species and each plant species increase from light blue to dark blue.
**Figure S7:** A, Bipartite network graph depicting the network analysis for the bee‐plant network at Cala d'Oliva in February–April. Link width and blue shading level indicates the frequency of visits to plant species by a given bee species. B, Modularity of the bee‐plant network at Cala d'Oliva in February–April. The *y*‐axis represents the plants and the *x*‐axis represents the bees. The red rectangles are the communities that emerged through modularity. The frequencies of interactions—where they differ from zero—between each bee species and each plant species increase from light blue to dark blue.
**Figure S8:** A, Bipartite network graph depicting the network analysis for the bee‐plant network at Cala d'Oliva in May–July. Link width and blue shading level indicates the frequency of visits to plant species by a given bee species. B, Modularity of the bee‐plant network at Cala d'Oliva in May–July. The *y*‐axis represents the plants and the *x*‐axis represents the bees. The red rectangles are the communities that emerged through modularity. The frequencies of interactions—where they differ from zero—between each bee species and each plant species increase from light blue to dark blue.
**Figure S9:** A, Bipartite network graph depicting the network analysis for the bee‐plant network at Fornelli in February–April. Link width and blue shading level indicates the frequency of visits to plant species by a given bee species. B, Modularity of the bee‐plant network at Fornelli in February–April. The *y*‐axis represents the plants and the *x*‐axis represents the bees. The red rectangles are the communities that emerged through modularity. The frequencies of interactions—where they differ from zero—between each bee species and each plant species increase from light blue to dark blue.
**Figure S10:** A, Bipartite network graph depicting the network analysis for the bee‐plant network at Fornelli in May–July. Link width and blue shading level indicates the frequency of visits to plant species by a given bee species. B, Modularity of the bee‐plant network at Fornelli in May–July. The *y*‐axis represents the plants and the *x*‐axis represents the bees. The red rectangles are the communities that emerged through modularity. The frequencies of interactions—where they differ from zero—between each bee species and each plant species increase from light blue to dark blue.
**Figure S11:** The *z‐*score (= (observed value—mean of null model values)/standard deviation of null model values) calculated for network indices in all studied networks. The index is significantly different from expected when the |*z‐*score| is > 1.96. F‐A: February–April, M‐J: May–July, A‐N: August–November.

ece373394‐sup‐0002‐DATASET_FlorSurv.xlsx.

ece373394‐sup‐0003‐DATASET_BeePlant.xlsx.

ece373394‐sup‐0004‐DATASET_SpeciesLevel.xlsx.

## Data Availability

The complete set of original data collected in this study is provided in the article and in the Supporting Information S1.
