# Supplementary material for: Unveiling the Patterns of Wild Bee‐Plant Interactions on a Large and Mostly Unexplored Mediterranean Island (Sardinia, Italy)
Source: Ecol Evol. 2026 Apr 6;16(4):e73394. doi: 10.1002/ece3.73394 (PMC13053173; doi:10.1002/ece3.73394)
Supplement: Supplementary file 1 — Table S1: Location and elevation of the four sampling sites (north‐western Sardinia, Italy). Table S2: Habitat composition (%) of the four sampling sites (north‐western Sardinia, Italy). Table S3: List of the wild bee species collected by standardized transect walks during 3 years of sampling (2021‐2022‐2023) across the seasons (February to November) and in the four sites of the study area (Northwest Sardinia, Italy). Table S4: Differences in the observed species richness (S), Shannon–Wiener diversity (H) and Gini‐Simpson dominance (GS) of the studied communities across periods. Table S5: Collection data on wild bee species identified as new records for Sardinia and, in the case of Hoplitis corsaria (Warncke, 1991), also for Italy. Table S6: List of plant species at the four sampling sites. The gray color of the cell indicates the presence of the plant species at that site, while the “×” signifies that the species has been visited by at least one wild bee. Figure S1: Temporal patterns of whole community plant diversity (as maximum number of species recorded per plot across 3 years‐period) from February (F) to November (N), at each of the four studied sites. Figure S2: Correlation matrix showing the linear relationships between all the vegetation‐associated and habitat‐associated variables considered in this study. The scale bar represents the Pearson correlation coefficient. Only the significant correlations (p < 0.05) are shown. Figure S3: Rarefaction and extrapolation curves for species diversity (richness) of bee species in the four sites. Shaded areas are 95% confidence intervals. Figure S4: A, Bipartite network graph depicting the network analysis for the bee‐plant network at Ottava in February–April. Link width and blue shading level indicates the frequency of visits to plant species by a given bee species. B, Modularity of the bee‐plant network at Ottava in February–April. The y‐axis represents the bees and the x‐axis represents the plants. The red rectangles [file ECE3-16-e73394-s002.pdf]

Supporting information for

Unveiling the patterns of wild bee-plant interactions on a large and mostly unexplored Mediterranean island (Sardinia, Italy)

Journal: Ecology and Evolution

Matteo Lezzeri[1], Vanessa Lozano[1,2], Stephane Knoll[1], Simone Flaminio[3], Giuseppe Brundu[1,2], Ignazio Floris[1,2], Michelina Pusceddu[1,2], Marino Quaranta[3], Carlo Polidori[4*], Alberto Satta[1,2*]

[1] Department of Agricultural Sciences, University of Sassari, Viale Italia 39/A, 07100 Sassari, Italy

[2] National Biodiversity Future Center (NBFC), Piazza Marina 61, 90133 Palermo, Italy

[3] CREA Research Centre for Agriculture and Environment (CREA-AA), Via Di Corticella 133, 40128 Bologna, Italy

[4] Department of Environmental Science and Policy (ESP), University of Milan, Via Celoria 26, 20133, Milan, Italy

***Corresponding authors:**

Carlo Polidori: carlo.polidori@unimi.it

Alberto Satta: albsatta@uniss.it

Table S1 Location and elevation of the four sampling sites (north-western Sardinia, Italy).

Sampling site		Geographic coordinates (WGS84) for each point of the transect		Mean elevation (m) above sea level (min and max)
		Latitude N	Longitude E	
Cala D'Oliva	A	41° 5' 3.77"	8° 20' 15.54"	17 (6-184)
	B	41° 5' 2.40"	8° 20' 14.82"	
	C	41° 5' 0.96"	8° 20' 15.50"	
	D	41° 5' 1.79"	8° 20' 16.55"	
	E	41° 5' 0.71"	8° 20' 17.16"	
Fornelli	A	40° 59' 55.43"	8° 14' 36.42"	38 (12-243)
	B	40° 59' 56.94"	8° 14' 35.74"	
	C	40° 59' 58.45"	8° 14' 35.99"	
	D	41° 0' 0.46"	8° 14' 35.48"	
	E	41° 0' 2.08"	8° 14' 35.46"	
Baratz	A	40° 41' 22.27"	8° 13' 14.21"	52 (25-87)
	B	40° 41' 20.89"	8° 13' 13.73"	
	C	40° 41' 21.06"	8° 13' 15.14"	
	D	40° 41' 22.48"	8° 13' 16.13"	
	E	40° 41' 23.30"	8° 13' 17.94"	
Ottava	A	40° 46' 18.74"	8° 29' 21.97"	71 (57-100)
	B	40° 46' 20.71"	8° 29' 19.83"	
	C	40° 46' 21.54"	8° 29' 17.36"	
	D	40° 46' 21.63"	8° 29' 19.83"	
	E	40° 46' 21.82"	8° 29' 21.69"	

Table S2 Habitat composition (%) of the four sampling sites (north-western Sardinia, Italy). EUNIS Code = habitat code according to the EUNIS habitat classification system (European Environment Agency, https://eunis.eea.europa.eu/habitats-names.jsp) and respective abridged description of the habitat.

Habitat type			Sampling site			
			Asinara		Nurra	
EUNIS Code	Abridged description	Natural (N), Semi-natural (SN), Agricultural-Crop (C), and Constructed-Urban (U) habitat	Cala d'Oliva	Fornelli	Baratz	Ottava
A	Marine habitats	N	33.56			
B1	Coastal dunes and sandy shores	N	0.05			
B3.3	Rock cliffs, ledges and shores, with angiosperms	N	3.71			
C1	Surface standing waters	N	0.01	0.40	5.27	
C3.32	*Arundo donax* beds	SN				1.41
C3.421	Short Mediterranean amphibious communities	N			1.73	
E1.313	Mediterranean annual communities of shallow soils	SN	4.84	9.91	19.98	
E3.2	Mediterranean short humid grassland	N		0.48		
F5.511	Oleo-lentisc brush	N-SN			37.57	17.06
F5.52	*Euphorbia dendroides* formations	SN	54.94	64.77		
FB.41	Traditional vineyards	C			0.34	0.06
G1.1	Riparian and gallery woodland, with dominant *Alnus*, *Betula*, *Populus* or *Salix*	N-SN	0.12			
G1.D	Fruit and nut tree orchards	C		0.01		5.25
G1.D3	*Prunus amygdalus* groves	C				1.05
G2.91	*Olea europaea* groves	C			0.04	12.43
G3.73	*Pinus pinea* stands	C	0.03	0.18	14.76	
G3.74	*Pinus halepensis* stands	C				0.80
G5.1	Lines of trees	C		0.06		1.33
G5.2	Small broadleaved deciduous anthropogenic woodlands	SN	0.08			0.03
G5.4	Small coniferous anthropogenic woodlands	SN				0.01
G5.85	Shrubby clearings	C			0.09	
H3.62	Sparsely vegetated weathered rock and outcrop habitats	N	0.23	22.71	0.24	
I	Regularly or recently cultivated agricultural.	C				0.55

	horticultural and domestic habitats					
I1.22	Small-scale market gardens and horticulture. including allotments	C				0.01
I1.3	Arable land with unmixed crops grown by low-intensity agricultural methods	C			18.97	43.95
J	Constructed. industrial and other artificial habitats	U				0.12
J1.2	Residential buildings of villages and urban peripheries	U				0.01
J1.4	Urban and suburban industrial and commercial sites still in active use	U			0.03	3.80
J2.1	Scattered residential buildings	U	1.03	0.54	0.10	2.81
J4.1	Disused road rail and other constructed hard-surfaced areas	U	0.84	0.49	0.78	0.82
J4.2	Road networks	U	0.55	0.45	0.06	1.79
J4.3	Rail networks	U				0.56
J4.6	Pavements and recreation areas	U			0.04	1.77
J4.7	Constructed parts of cemeteries	U	0.01			
J5	Highly artificial man-made waters and associated structures	U				0.02
X25	Domestic gardens of villages and urban peripheries	U				4.37

Table S3 List of the wild bee species collected by standardized transect walks during three years of sampling (2021-2022-2023) across the seasons (February to November) and in the four sites of the study area (Northwest Sardinia, Italy).

**Species**	**Cala d'Oliva**	**Fornelli**	**Baratz**	**Ottava**
**Colletidae**				
Colletes hederae Schmidt & Westrich, 1993	0	0	3	3
Colletes similis Schenck, 1853	0	0	1	1
Hylaeus brevicornis Nylander, 1852 (*brevicornis* complex)	0	0	2	2
Hylaeus clypearis (Schenck, 1853)	0	0	0	14
Hylaeus communis Nylander, 1852	0	0	3	0
Hylaeus dilatatus (Kirby, 1802)	0	0	0	1
Hylaeus gibbus (Saunders, 1850) (*gibbus* complex)	0	0	1	0
Hylaeus imparilis Förster, 1871 (*brevicornis* complex)	0	0	1	1
Hylaeus taeniolatus Förster, 1871	0	3	0	1
Hylaeus variegatus (Fabricius, 1798)	0	0	0	1
**Andrenidae**				
Andrena agilissima (Scopoli, 1770)	7	3	3	1
Andrena alfkenella Perkins, 1914	0	0	1	2
Andrena cinerea Brullé, 1832	1	2	4	2
Andrena colletiformis Morawitz, 1874	0	0	0	4
Andrena compta Lepeletier, 1841	0	0	2	4
Andrena dorsata (Kirby, 1802) *	2	1	0	0
Andrena ferrugineicrus Dours, 1872	0	0	0	10
Andrena flavipes Panzer, 1799	14	9	20	66
Andrena floricola Eversmann, 1852	0	0	1	5
Andrena fulvago (Christ, 1791)	0	0	2	0
Andrena hesperia Smith, 1853	5	5	29	24
Andrena impunctata Pérez, 1895	0	0	0	3
Andrena lepida Schenck, 1859	3	49	0	0
Andrena minutula (Kirby, 1802)	1	0	11	9
Andrena morio Brullé, 1832	1	3	1	2
Andrena nana (Kirby, 1802)	3	0	0	0
Andrena nigroaenea (Kirby, 1802)	23	17	8	2
Andrena nigroolivacea Dours, 1873	0	0	2	53
Andrena cf *niveata* Friese, 1887	0	0	0	1
Andrena antonellae Praz & Genoud, 2022	0	0	0	1
Andrena ovatula (Kirby, 1802)	1	0	0	0
Andrena pilipes Fabricius, 1781	5	0	0	0
Andrena sardoa Lepeletier, 1841	3	4	0	0

Andrena simontornyella Noskiewicz, 1930	1	0	0	0
Andrena tenuistriata Pérez, 1895	0	0	1	0
Andrena thoracica (Fabricius, 1775)	0	1	5	5
Andrena vetula Lepeletier, 1841	6	1	7	2
Panurgus corsicus Warncke, 1972	19	9	4	10
Panurgus dentipes Latreille, 1811	13	8	4	10
**Halictidae**				
Halictus brunnescens (Eversmann, 1852)	0	0	1	0
Halictus fulvipes (Klug, 1817)	1	2	0	2
Halictus quadricinctus (Fabricius, 1776)	0	0	0	2
Halictus scabiosae (Rossi, 1790)	0	2	0	2
Lasioglossum albocinctum (Lucas, 1846)	2	1	1	6
Lasioglossum griseolum (Morawitz, 1872)	1	3	0	3
Lasioglossum inexpectatum Flaminio & Pauly, 2024	0	0	15	0
Lasioglossum interruptum (Panzer, 1798)	4	0	2	5
Lasioglossum lativentre (Schenck, 1853)	0	1	0	22
Lasioglossum malachurum (Kirby, 1802)	0	0	1	2
Lasioglossum marginatum (Brullé, 1832) *****	0	0	1	0
Lasioglossum medinai (Vachal, 1895) *****	0	0	1	1
Lasioglossum nigripes (Lepeletier, 1841)	0	0	3	0
Lasioglossum pauperatum (Brullé, 1832)	2	2	8	8
Lasioglossum pauxillum (Schenck, 1853)	3	0	5	7
Lasioglossum puncticolle (Morawitz, 1872)	0	0	0	1
Lasioglossum villosulum (Kirby, 1802)	0	0	0	4
Lasioglossum zonulum (Smith, 1848)	0	1	0	0
Seladonia gemmea (Dours, 1872)	1	7	5	5
Seladonia smaragdula (Vachal, 1895) (*smaragdula* complex)	0	0	1	3
Seladonia subaurata (Rossi, 1792)	0	0	0	1
Seladonia vestita Lepeletier, 1841	0	0	1	0
Sphecodes gibbus (Linnaeus, 1758)	0	0	0	2
**Melittidae**				
Dasypoda hirtipes (Fabricius, 1793)	0	0	2	0
**Megachilidae**				
Anthidium florentinum (Fabricius, 1775)	0	0	0	4
Anthidium manicatum (Linnaeus, 1758)	0	0	0	2
Heriades crenulata Nylander, 1856	0	1	0	2
Heriades rubicola Pérez, 1890	0	0	0	1

Heriades truncorum (Linnaeus, 1758)	0	1	0	1
Hoplitis adunca (Panzer, 1798)	3	0	0	0
Hoplitis benoisti (Alfken, 1935)	1	0	0	1
Hoplitis corsaria (Warncke, 1991) **	11	0	0	3
Hoplitis leucomelana (Kirby, 1802)	0	0	0	4
Lithurgus chrysurus Fonscolombe, 1834	5	1	0	0
Megachile albisecta (Klug, 1817)	0	0	4	1
Megachile argentata (Fabricius, 1793)	1	1	2	5
Megachile leachella Curtis, 1828	0	0	2	0
Megachile melanopyga A. Costa, 1863	0	0	1	2
Megachile parietina (Geoffroy, 1785)	0	0	0	1
Megachile sicula (Rossi, 1792)	0	1	0	0
Osmia andrenoides (Spinola, 1808)	0	0	0	3
Osmia bicornis (Linnaeus, 1758)	0	2	0	0
Osmia caerulescens (Linnaeus, 1758)	2	0	0	4
Osmia cornuta (Latreille, 1805)	0	2	0	0
Osmia latreillei (Spinola, 1806)	1	0	0	0
Osmia leaiana (Kirby, 1802) *	0	0	0	1
Osmia ligurica (Morawitz, 1868)	3	5	2	1
Osmia niveata (Fabricius, 1804)	0	0	1	0
Osmia notata (Fabricius, 1804)	1	0	0	0
Osmia rufohirta (Latreille, 1811)	0	0	0	12
Osmia scutellaris (Morawitz, 1868) *	1	3	1	3
Osmia submicans Morawitz, 1866	2	0	0	0
Pseudoanthidium melanurum (Klug, 1832)	1	0	0	2
Pseudonthidium nanum (Mocsáry, 1879)	1	1	0	1
Rhodanthidium septemdentatum (Latreille, 1809)	0	0	0	3
Trachusa interrupta (Fabricius, 1781)	0	0	10	1
**Apidae**				
Amegilla garrula (Rossi, 1790)	0	0	1	4
Anthophora canescens Brullé, 1832	0	1	0	0
Anthophora dispar Lepeletier, 1841	0	5	0	0
Anthophora nigrovittata Dours, 1869	28	9	0	0
Anthophora sichelii Radoszkowski, 1868	3	0	0	0
Bombus hortorum (Linnaeus, 1761)	0	0	2	2
Bombus terrestris (Linnaeus, 1758)	100	40	25	11
Ceratina cucurbitina (Rossi, 1792)	2	9	33	117
Ceratina cyanea (Kirby, 1802)	0	0	5	26
Ceratina dallatorreana Friese, 1896	0	1	2	50

Ceratina dentiventris Gerstaecker, 1869	0	0	0	2
Ceratina parvula Smith, 1854	0	0	0	1
Eucera clypeata Erichson, 1835	0	0	0	10
Eucera grisea Fabricius, 1793	0	0	0	3
Eucera nigrescens Pérez, 1879	2	0	4	0
Eucera nigrifacies Lepeletier, 1841	1	2	0	7
Eucera nigrilabris Lepeletier, 1841	0	6	1	31
Eucera numida Lepeletier, 1841	1	0	1	2
Eucera oraniensis Lepeletier, 1841	10	5	0	27
Nomada distinguenda Morawitz, 1874	0	0	1	0
Nomada femoralis Morawitz, 1869	0	0	0	3
Nomada flavoguttata (Kirby, 1802) *****	0	0	0	2
Nomada kholi Schmiedeknecht, 1882	0	0	0	1
Tetralonia dentata (Klug, 1835)	4	7	1	0
Thyreyus histrionicus (Illiger. 1806)	0	0	1	0
Xylocopa iris (Christ, 1791)	0	0	1	0
Xylocopa violacea (Linnaeus, 1758)	5	1	5	0
**Total number of species**	**47**	**43**	**59**	**84**
**Total number of individuals collected**	**311**	**238**	**269**	**673**

* New species for Sardinia; ** New species for Italy

Table S4. Differences in the observed species richness (*S*), Shannon-Wiener diversity (*H*) and Gini-Simpson dominance (*GS*) of the studied communities across periods.

Site	Index	*P* (Feb-Apr vs. May-July)	*P* (May-July vs. Aug-Nov)	*P* (Feb-Apr vs. Aug-Nov)
**Bees**				
**Baratz**	*S*	0.003	0.001	0.188
	N	0.000	0.000	0.000
	H	0.000	0.000	0.051
	GS	0.000	0.000	0.015
**Ottava**	*S*	0.000	0.210	1.000
	N	0.000	0.000	0.000
	H	0.000	0.137	0.845
	GS	0.006	0.213	0.528
**Cala d'Oliva**	*S*	0.979	-	-
	N	0.000	-	-
	H	0.003	-	-
	GS	0.000	-	-
**Fornelli**	*S*	0.670	-	-
	N	0.000	-	-
	H	0.844	-	-
	GS	0.310	-	-
**Plants (network)**				
**Baratz**	*S*	0.239	0.000	0.037
	N	0.000	0.000	0.000
	H	0.000	0.000	0.087
	GS	0.000	0.000	0.929
**Ottava**	*S*	0.220	0.089	0.962
	N	0.000	0.000	0.000
	H	0.000	0.005	0.347
	GS	0.000	0.004	0.065
**Cala d'Oliva**	*S*	0.010	-	-

	N	0.000	-	-
	H	0.000	-	-
	GS	0.000	-	-
**Fornelli**	*S*	0.939	-	-
	N	0.000	-	-
	H	0.323	-	-
	GS	0.063	-	-
**Plants (community)**				
**Baratz**	*S*	0.385	0.000	0.000
	N	0.000	0.000	0.000
	H	0.000	0.000	0.000
	GS	0.000	0.000	0.000
**Ottava**	*S*	0.000	0.00	0.00
	N	0.000	0.00	0.00
	H	0.000	0.00	0.00
	GS	0.000	0.00	0.00
**Cala d'Oliva**	*S*	0.00	-	-
	N	0.00	-	-
	H	0.00	-	-
	GS	0.00	-	-
**Fornelli**	*S*	0.000	-	-
	N	0.000	-	-
	H	0.000	-	-
	GS	0.000	-	-

Table S5. Collection data on wild bee species identified as new records for Sardinia and, in the case of *Hoplitis corsaria* (Warncke, 1991), also for Italy.

Species	Site	♀	♂	Collection date	Leg.
Andrena dorsata (Kirby, 1802)	Cala d'Oliva	2		11.IV.2023	M. Gabaglio
	Fornelli	1		7.IV.2023	S. Knoll
Hoplitis corsaria (Warncke, 1991)	Cala d'Oliva		2	27.V.2021	M. Lezzeri
	Cala d'Oliva		1	29.IV.2022	M. Lezzeri
	Cala d'Oliva	1	4	16.V.2022	M. Lezzeri
	Cala d'Oliva		2	5.V.2023	M. Gabaglio
	Cala d'Oliva	1		5.V.2023	S. Knoll
	Ottava		3	23.V.2022	M. Lezzeri
	Ottava	9		12.IV.2023	M. Gabaglio
Lasioglossum marginatum (Brullé, 1832)	Baratz	1		28.VI.2023	S. Knoll
Lasioglossum medinai (Vachal, 1895)	Baratz	1		25.VII.2023	S. Knoll
	Ottava	1		14.IX.2023	M. Gabaglio
Osmia leaiana (Kirby, 1802)	Ottava		1	23.V.2022	M. Lezzeri
Osmia scutellaris (Morawitz, 1868)	Baratz		1	10.V.2023	S. Knoll
	Ottava	2	1	16.V.2023	S. Knoll
	Fornelli	3		5.V.2023	S. Knoll
	Cala d'Oliva		1	5.V.2023	S. Knoll
Nomada flavoguttata (Kirby, 1802)	Ottava	2		12.IV.2023	M. Gabaglio

Table S6 List of plant species at the four sampling sites. The gray color of the cell indicates the presence of the plant species at that site, while the ‘x’ signifies that the species has been visited by at least one wild bee.

Species	Baratz	Ottava	Fornelli	Cala d'Oliva
Ajuga iva (L.) Schreb.				
Allium roseum L. subsp. *roseum*				
Allium subhirsutum L. subsp. *subhirsutum*				
Allium triquetrum L.	x			
Ammi majus L.				
Andryala integrifolia L.			x	
Anemone hortensis L.				
Anthemis arvensis L.			x	x
Arisarum vulgare O.Targ.Tozz. subsp. *vulgare*				
Artemisia arborescens (Vaill.) L.				
Arum italicum Mill. subsp. *italicum*				
Asparagus acutifolius L.				
Asphodelus ramosus L. subsp. *ramosus*	x		x	x
Astragalus pelecinus (L.) Barneby subsp. *pelecinus*				
Ballota nigra L.		x		
Bellardia trixago (L.) All.				
Bellardia viscosa (L.) Fisch. & C.A.Mey.				
Bellis annua L.				
Bellis perennis L.		x		
Borago officinalis L.		x		
Bryonia marmorata E.Petit				
Bunias erucago L.				x
Calendula arvensis (Vaill.) L.	x	x	x	x
Capsella bursa-pastoris (L.) Medik. subsp. *bursa-pastoris*		x		
Cardamine hirsuta L.				
Carduus pycnocephalus L. subsp. *pycnocephalus*	x			
Carlina corymbosa L.	x		x	
Carlina lanata L.				
Carthamus lanatus L.				
Centaurea calcitrapa L.		x		
Centaurea diluta Aiton		x		
Centaurium erythraea Rafn				
Centaurium maritimum (L.) Fritsch				
Centaurium pulchellum (Sw.) Hayek ex Hand.-Mazz., Stadlm., Janch. & Faltis subsp. *pulchellum*				
Centaurium tenuiflorum (Hoffmanns. & Link) Fritsch				
Centranthus calcitrapae (L.) Dufr. subsp. *calcitrapae*				
Cerastium glomeratum Thuill.				
Cerinthe major L. subsp. *major*		x		
Chenopodium album L.				
Cichorium intybus L.	x	x		
Cistus monspeliensis L.	x		x	x

Clinopodium nepeta (L.) Kuntze		x		
Coleostephus myconis (L.) Cass. ex Rchb.f.			x	
Convolvulus arvensis L.	x	x		
Convolvulus cantabrica L.				
Crepis foetida L. subsp. *foetida*				
Crepis vesicaria L.	x	x		x
Cynara cardunculus L.	x			
Cynoglossum creticum Mill.				
Daucus carota L.	x	x		
Dioscorea communis (L.) Caddick & Wilkin				
Diplotaxis tenuifolia (L.) DC.				
Dittrichia viscosa (L.) Greuter	x			
Echium plantagineum L.	x	x	x	x
Erigeron bonarensis L.		x		
Erodium moschatum (L.) L'Hér.		x	x	
Eruca vesicaria (L.) Cav.		x		
Eryngium campestre L.				
Euphorbia dendroides L.			x	x
Euphorbia helioscopia L. subsp. *helioscopia*	x			
Euphorbia pithyusa L.	x			
Ferula communis L. subsp. *communis*				x
Ficaria verna Huds.				
Foeniculum vulgare Mill.		x		
Fumaria bicolor Sommier				
Fumaria capreolata L.				
Fumaria officinalis L. subsp. *officinalis*	x	x		
Galactites tomentosus Moench	x	x	x	x
Galium aparine L.				
Geranium dissectum L.				
Geranium molle L.		x		x
Geranium robertianum L.				
Glebionis coronaria (L.) Spach	x	x	x	x
Glebionis segetum (L.) Fourr.			x	
Hedypnois rhagadioloides (L.) F.W.Schmidt	x			
Heliotropium europaeum L.		x		
Helminthotheca echioides (L.) Holub	x			
Hyoseris radiata L.		x		x
Hypericum perforatum L.				
Hypochaeris achyrophorus L.	x	x	x	x
Hypochaeris glabra L.	x			
Hypochaeris radicata L.	x	x		
Jacobaea delphiniifolia (Vahl) Pelser & Veldkamp		x		
Jacobaea erucifolia (L.) G.Gaertn., B.Mey. & Scherb.		x		
Kickxia commutata (Bernh. ex Rchb.) Fritsch				
Lactuca virosa L.				
Lamium amplexicaule L.				
Lathyrus cicera L.				

Lathyrus clymenum L.				
Lathyrus ochrus (L.) DC.				
Lathyrus oleraceus Lam.				
Linum strictum L.				
Linum trigynum L.				
Linum usitatissimum L. subsp. *angustifolium*				
Lobularia maritima (L.) Desv.				
Logfia gallica (L.) Cosson & Germ.				
Lotus edulis L.				
Lotus ornithopodioides L.		x		x
Lotus tetragonolobus L.				
Lysimachia arvensis (L.) U.Manns & Anderb.	x	x	x	x
Lythrum hyssopifolia L.				
Malva multiflora (Cav.) Soldano, Banfi & Galasso	x	x		
Malva sylvestris L.				x
Medicago arabica (L.) Huds.				
Medicago coronata (L.) Bartal.				
Medicago littoralis Rohde ex Loisel.				
Medicago minima (L.) L.				
Medicago murex Willd.				
Medicago orbicularis (L.) Bartal.				
Medicago polymorpha L.		x		
Medicago rugosa Desr.				
Medicago truncatula Gaertn.				
Mentha pulegium L. subsp. *pulegium*		x		
Mercurialis annua L.				
Misopates orontium (L.) Raf.				
Myosotis arvensis (L.) Hill		x		
Nigella damascena L.				
Oenanthe crocata L.				
Olea europaea L.				
Ononis spinosa L. subsp. *spinosa*				
Onopordum horridum Viv.				x
Ophrys panormitana (Tod.) Soó				
Ornithopus compressus L.				
Oxalis pes-caprae L.	x			
Pallenis spinosa (L.) Cass.	x	x		
Papaver hybridum L.				
Papaver rhoeas L.				
Parietaria judaica L.				
Paronychia echinulata Chater				
Petrorhagia dubia (Raf.) G.López & Romo				
Petrorhagia prolifera (L.) P.W.Ball & Heywood				
Pinus halepensis Mill.				
Pistacia lentiscus L.				x
Plantago afra L.				
Plantago lagopus L.	x	x	x	x

Plantago macrorhiza Poir.				
Plantago major L.				
Polygonum aviculare L. subsp. *aviculare*				
Potentilla reptans L.				
Quercus ilex L.				
Ranunculus muricatus L.				
Raphanus raphanistrum L. subsp. *raphanistrum*	x	x	x	x
Raphanus raphanistrum L. subsp. *sativus* (L.) Schmalh.	x	x		x
Rapistrum rugosum (L.) All.				
Reichardia picroides (L.) Roth	x			
Reseda alba L.		x		
Rhamnus alaternus L.		x		
Rubia peregrina L.				
Rubus ulmifolius Schott	x	x		
Rumex bucephalophorus L.				
Ruta chalepensis L.				
Salvia clandestina L.				
Salvia verbenaca L.				
Scorpiurus muricatus L.				
Scrophularia peregrina L.				
Senecio vulgaris L.		x		
Sherardia arvensis L.				
Silene gallica L.				x
Silene latifolia Poir.		x		
Sinapis alba L.		x		x
Sinapis arvensis L. subsp. *arvensis*		x		x
Sisymbrium officinale (L.) Scop.				x
Sixalix atropurpurea (L.) Greuter & Burdet	x	x		
Smilax asper L.				
Smyrnium olusatrum L.				
Solanum nigrum L.				
Sonchus asper (L.) Hill				
Sonchus oleraceus L.		x		
Stachys arvensis (L.) L.				
Stachys major (L.) Bartolucci & Peruzzi				
Stachys romana (L.) E.H.L.Krause		x		
Stellaria media (L.) Vill.		x		
Tamarix gallica L.				
Tolpis umbellata Bertol.			x	
Torilis arvensis (Huds.) Link				
Trifolium alexandrinum L.				
Trifolium angustifolium L. subsp. *angustifolium*				
Trifolium arvense L. subsp. *arvense*				
Trifolium campestre Schreb.				
Trifolium glomeratum L.		x		
Trifolium nigrescens Viv. subsp. *nigrescens*		x	x	x
Trifolium repens L.		x		

Trifolium squarrosum L.				
Trifolium stellatum L.				
Trifolium tomentosum L.		x		
Trigonella officinalis (L.) Coulot & Rabaute				
Trigonella sicula (Turra) Coulot & Rabaute				
Urospermum dalechampii (L.) Scop. ex F.W.Schmidt				
Urospermum picroides (L.) Scop. ex F.W.Schmidt				
Verbascum sinuatum L.				
Verbena officinalis L.		x		
Veronica cymbalaria Bodard				
Veronica persica Poir.				x
Vicia benghalensis L.				
Vicia lathyroides L.				
Vicia sativa L.				

Supplementary figures

Figure S1. Temporal patterns of whole community plant diversity (as maximum number of species recorded *per* plot across three year-period) from February (F) to November (N), at each of the four studied sites.

Figure S2. Correlation matrix showing the linear relationships between all the vegetation-associated and habitat-associated variables considered in this study. The scale bar represents the Pearson correlation coefficient. Only the significant correlations ($P < 0.05$) are shown.

Figure S3. Rarefaction and extrapolation curves for species diversity (richness) of bee species in the four sites. Shaded areas are 95% confidence intervals.

Figure S4. A, Bipartite network graph depicting the network analysis for the bee-plant network at Ottava in February-April. Link width and blue shading level indicates the frequency of visits to plant species by a given bee species. B, Modularity of the bee-plant network at Ottava in February-April. The y-axis represents the plants and the x-axis represents the bees. The red rectangles are the communities that emerged through modularity. The frequencies of interactions – where they differ from zero – between each bee species and each plant species increase from light blue to dark blue.

Figure S5. A, Bipartite network graph depicting the network analysis for the bee-plant network at Ottava in May-July. Link width and blue shading level indicates the frequency of visits to plant species by a given bee species. B, Modularity of the bee-plant network at Ottava in May-July. The y-axis represents the plants and the x-axis represents the bees. The red rectangles are the communities that emerged through modularity. The frequencies of interactions – where they differ from zero – between each bee species and each plant species increase from light blue to dark blue.

Figure S6. A, Bipartite network graph depicting the network analysis for the bee-plant network at Ottava in August-November. Link width and blue shading level indicates the frequency of visits to plant species by a given bee species. B, Modularity of the bee-plant network at Ottava in August-November. The y-axis represents the plants and the x-axis represents the bees. The red rectangles are the communities that emerged through modularity. The frequencies of interactions – where they differ from zero – between each bee species and each plant species increase from light blue to dark blue.

Figure S7. A, Bipartite network graph depicting the network analysis for the bee-plant network at Cala d'Oliva in February-April. Link width and blue shading level indicates the frequency of visits to plant species by a given bee species. B, Modularity of the bee-plant network at Cala d'Oliva in February-April. The y-axis represents the plants and the x-axis represents the bees. The red rectangles are the communities that emerged through modularity. The frequencies of interactions – where they

differ from zero – between each bee species and each plant species increase from light blue to dark blue.

Figure S8. A, Bipartite network graph depicting the network analysis for the bee-plant network at Cala d'Oliva in May-July. Link width and blue shading level indicates the frequency of visits to plant species by a given bee species. B, Modularity of the bee-plant network at Cala d'Oliva in May-July. The y-axis represents the plants and the x-axis represents the bees. The red rectangles are the communities that emerged through modularity. The frequencies of interactions – where they differ from zero – between each bee species and each plant species increase from light blue to dark blue.

Figure S9. A, Bipartite network graph depicting the network analysis for the bee-plant network at Fornelli in February-April. Link width and blue shading level indicates the frequency of visits to plant species by a given bee species. B, Modularity of the bee-plant network at Fornelli in February-April. The y-axis represents the plants and the x-axis represents the bees. The red rectangles are the communities that emerged through modularity. The frequencies of interactions – where they differ from zero – between each bee species and each plant species increase from light blue to dark blue.

Figure S10. A, Bipartite network graph depicting the network analysis for the bee-plant network at Fornelli in May-July. Link width and blue shading level indicates the frequency of visits to plant species by a given bee species. B, Modularity of the bee-plant network at Fornelli in May-July. The y-axis represents the plants and the x-axis represents the bees. The red rectangles are the communities that emerged through modularity. The frequencies of interactions – where they differ from zero – between each bee species and each plant species increase from light blue to dark blue.

Figure S11. The *z*-score (= (observed value – mean of null model values)/standard deviation of null model values) calculated for network indices in all studied networks. The index is significantly different from expected when the |z-score| is > 1.96. F-A: February-April, M-J: May-July, A-N: August-November.

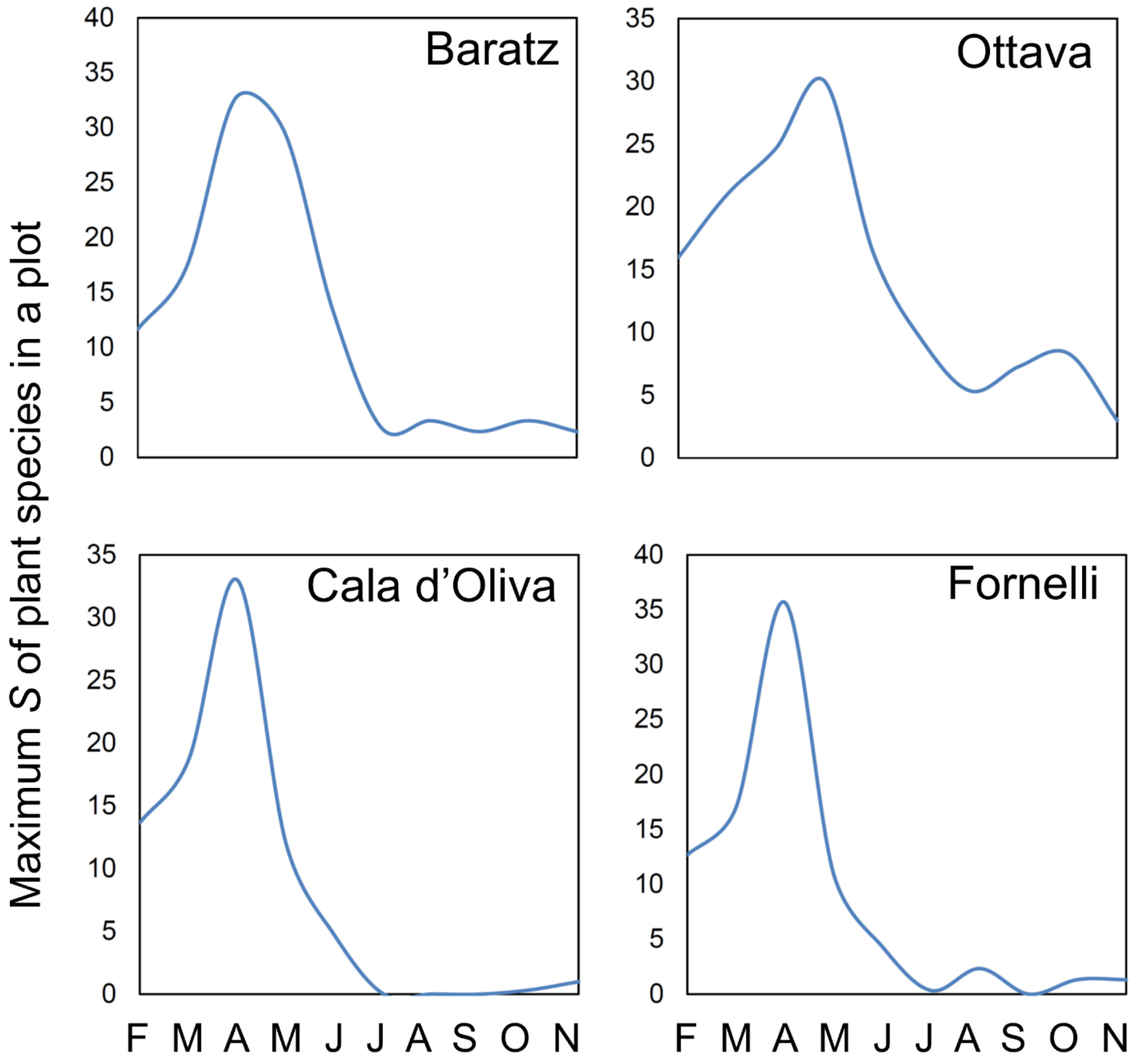
Maximum S of plant species in a plot
Baratz
Ottava
Cala d'Oliva
Fornelli
F M A M J J A S O N
F M A M J J A S O N
40
35
30
25
20
15
10
5
0

Figure S1

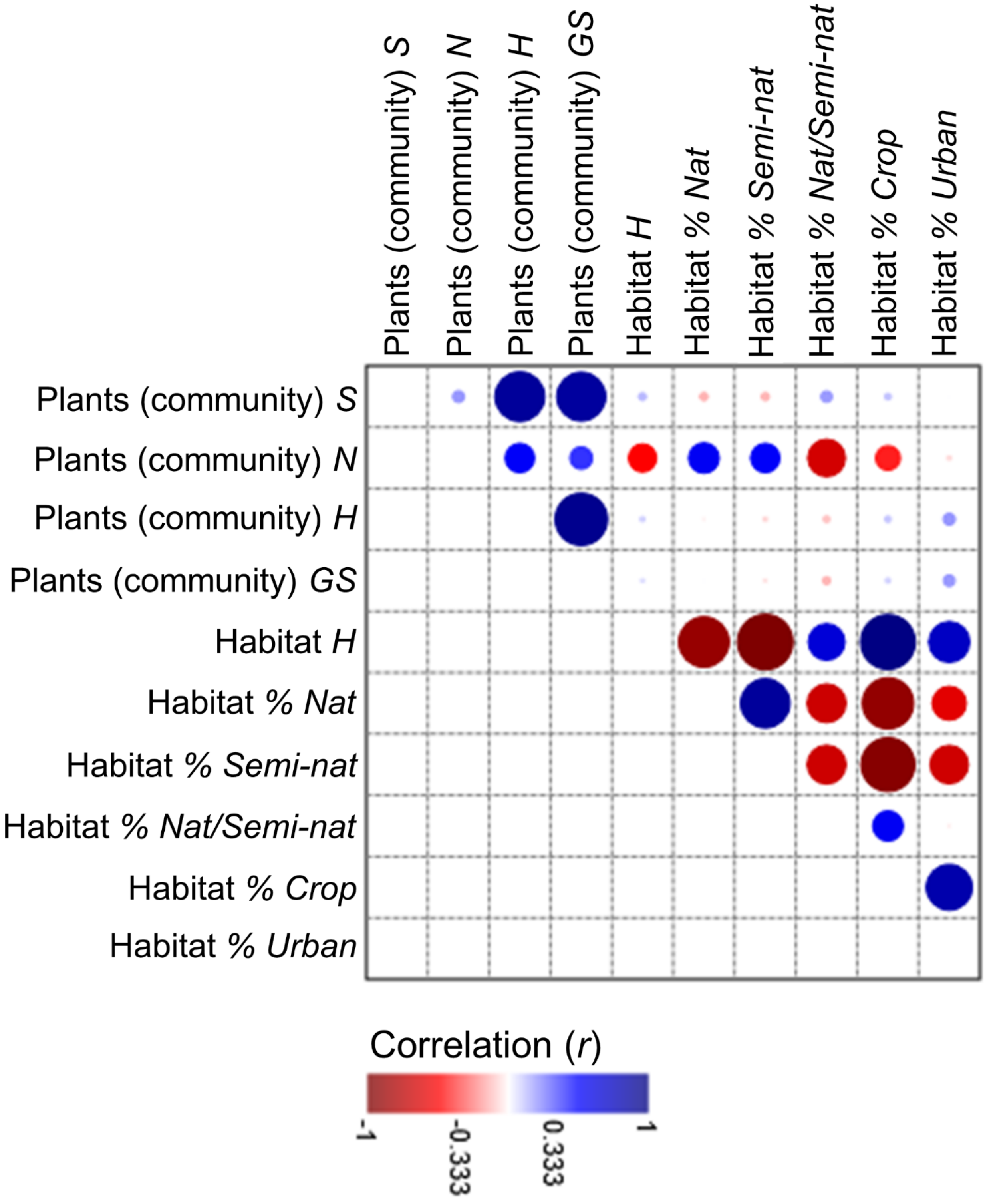
Plants (community) S
Plants (community) N
Plants (community) H
Plants (community) GS
Habitat H
Habitat % Nat
Habitat % Semi-nat
Habitat % Nat/Semi-nat
Habitat % Crop
Habitat % Urban
Correlation (r)
-1
-0.333
0.333
1

Figure S2

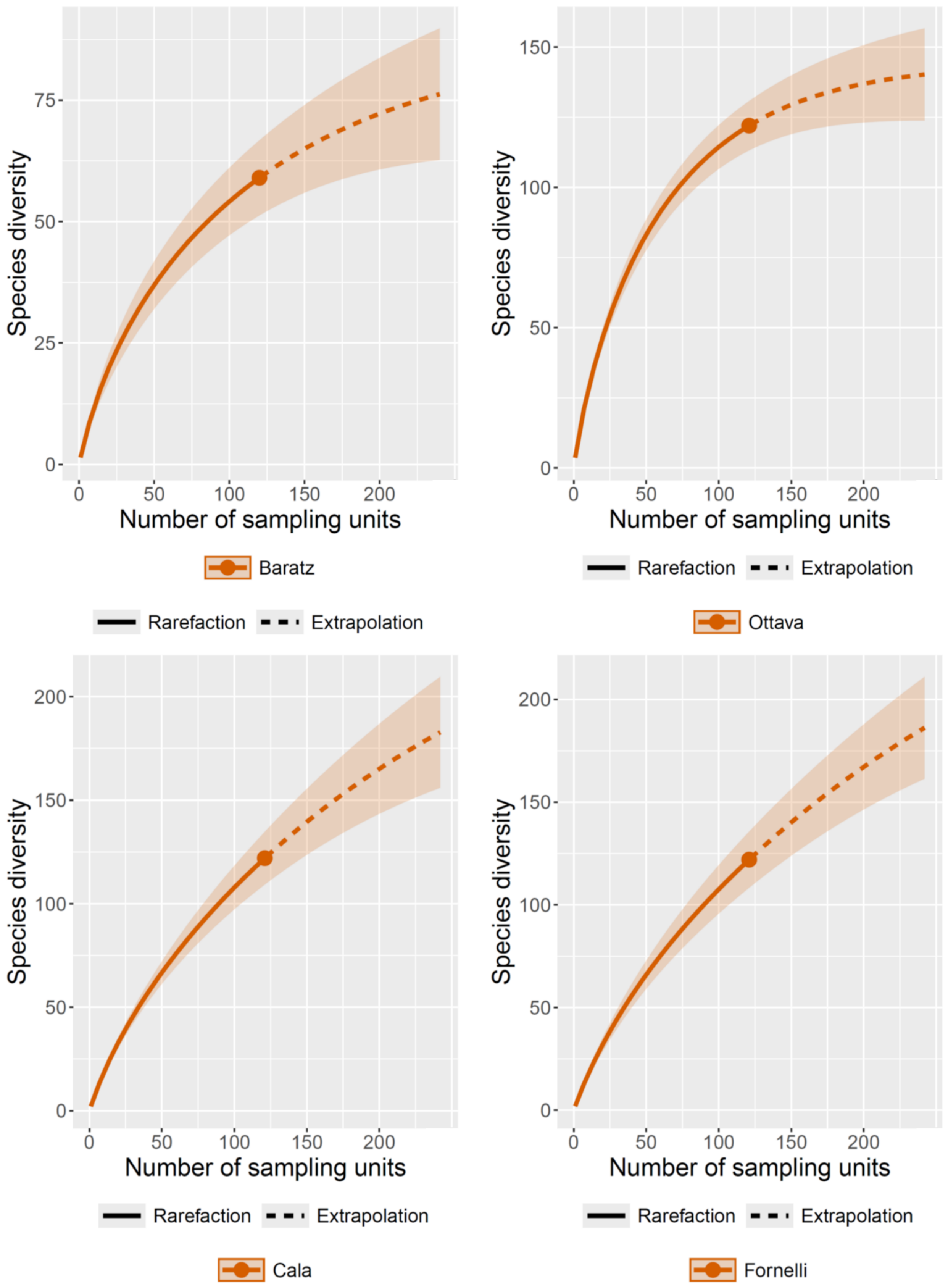
Species diversity
Number of sampling units
Baratz
Rarefaction
Extrapolation
Ottava
Cala
Fornelli
0
25
50
75
100
150
200

Figure S3

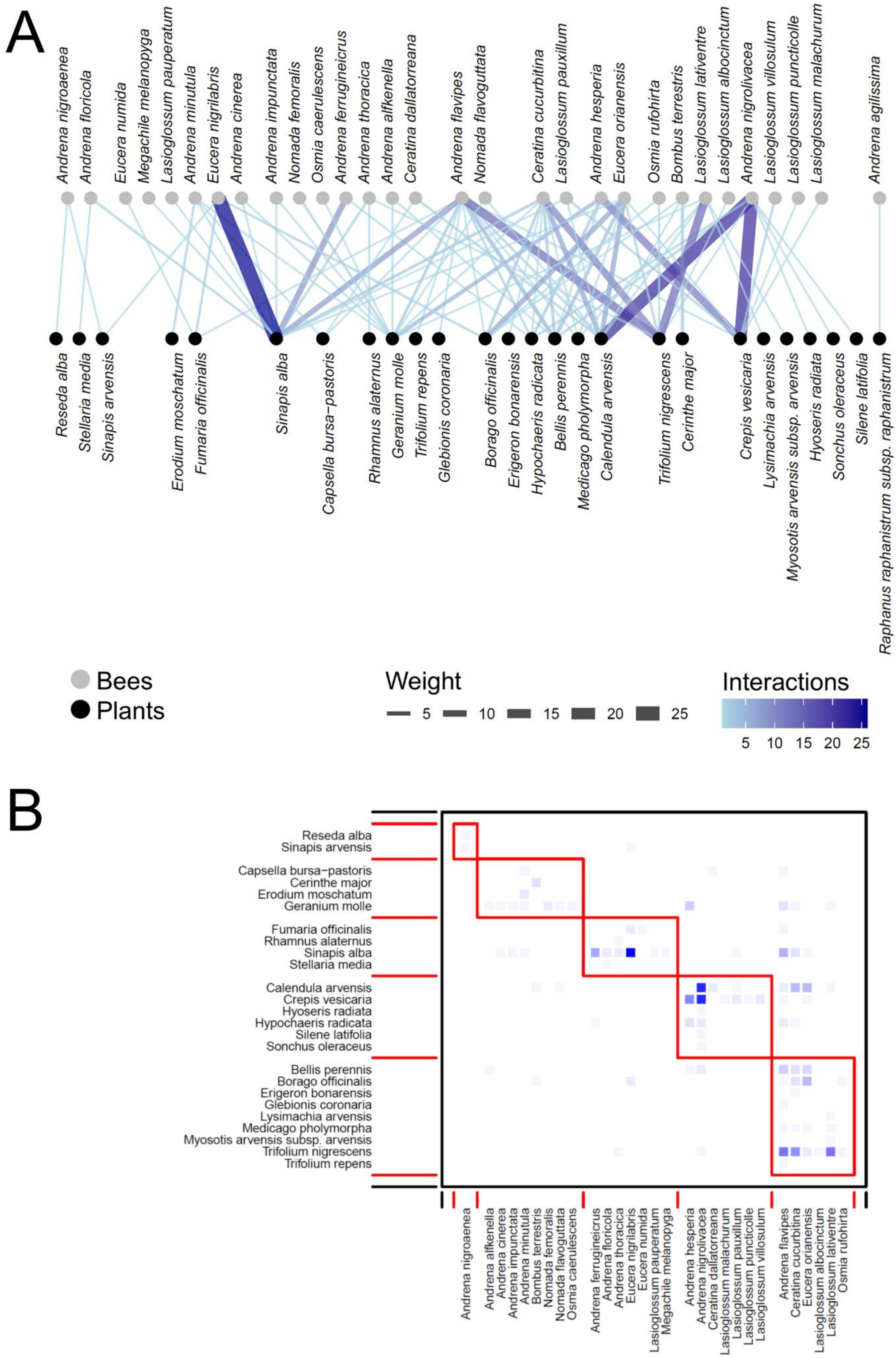
A
Andrena nigroaenea
Andrena floricola
Eucera numida
Megachile melanopyga
Lasioglossum pauperatum
Andrena minutula
Eucera nigrilabris
Andrena cinerea
Andrena impunctata
Nomada femoralis
Osmia caerulescens
Andrena ferrugineicrus
Andrena thoracica
Andrena alfkenella
Ceratina dallatorreana
Andrena flavipes
Nomada flavoguttata
Ceratina cucurbitina
Lasioglossum pauxillum
Andrena hesperia
Eucera oraniensis
Osmia rufohirta
Bombus terrestris
Lasioglossum lativentre
Lasioglossum albocinctum
Andrena nigrolivacea
Lasioglossum villosulum
Lasioglossum puncticolle
Lasioglossum malachurum
Andrena agilissima
Reseda alba
Stellaria media
Sinapis arvensis
Erodium moschatum
Fumaria officinalis
Sinapis alba
Capsella bursa−pastoris
Rhamnus alaternus
Geranium molle
Trifolium repens
Glebionis coronaria
Borago officinalis
Erigeron bonarensis
Hypochaeris radicata
Bellis perennis
Medicago pholymorpha
Calendula arvensis
Trifolium nigrescens
Cerinthe major
Crepis vesicaria
Lysimachia arvensis
Myosotis arvensis subsp. arvensis
Hyoseris radiata
Sonchus oleraceus
Silene latifolia
Raphanus raphanistrum subsp. raphanistrum
Bees
Plants
Weight
5
10
15
20
25
Interactions
5
10
15
20
25
B
Reseda alba
Sinapis arvensis
Capsella bursa−pastoris
Cerinthe major
Erodium moschatum
Geranium molle
Fumaria officinalis
Rhamnus alaternus
Sinapis alba
Stellaria media
Calendula arvensis
Crepis vesicaria
Hyoseris radiata
Hypochaeris radicata
Silene latifolia
Sonchus oleraceus
Bellis perennis
Borago officinalis
Erigeron bonarensis
Glebionis coronaria
Lysimachia arvensis
Medicago pholymorpha
Myosotis arvensis subsp. arvensis
Trifolium nigrescens
Trifolium repens
Andrena nigroaenea
Andrena alfkenella
Andrena cinerea
Andrena impunctata
Andrena minutula
Bombus terrestris
Nomada femoralis
Nomada flavoguttata
Osmia caerulescens
Andrena ferrugineicrus
Andrena floricola
Andrena thoracica
Eucera nigrilabris
Eucera numida
Lasioglossum pauperatum
Megachile melanopyga
Andrena hesperia
Andrena nigrolivacea
Ceratina dallatorreana
Lasioglossum malachurum
Lasioglossum pauxillum
Lasioglossum puncticolle
Lasioglossum villosulum
Andrena flavipes
Ceratina cucurbitina
Eucera oraniensis
Lasioglossum albocinctum
Lasioglossum lativentre
Osmia rufohirta

Figure S4

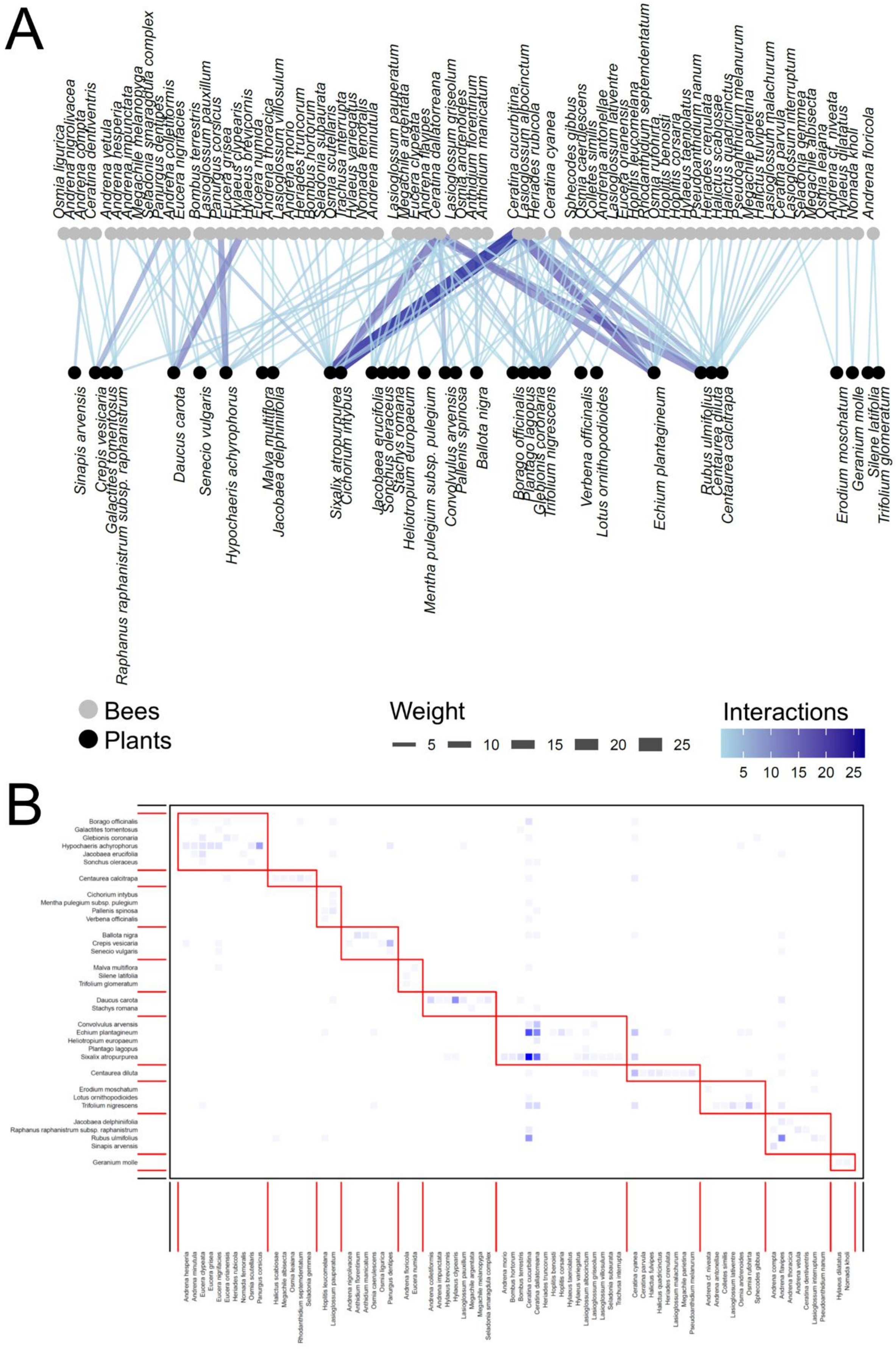
A
Osmia ligurica
Andrena nigrolivacea
Andrena compta
Ceratina dentiventris
Andrena vetula
Andrena hesperia
Andrena impunctata
Megachile melanopyga
Seladonia smaragdula complex
Panurgus dentipes
Andrena colletiformis
Eucera nigrifacies
Bombus terrestris
Lasioglossum pauxillum
Panurgus corsicus
Eucera grisea
Hylaeus clypearis
Hylaeus brevicornis
Eucera numida
Andrena thoracica
Lasioglossum villosulum
Andrena morio
Heriades truncorum
Bombus hortorum
Seladonia subaurata
Osmia scutellaris
Trachusa interrupta
Hylaeus variegatus
Nomada femoralis
Andrena minutula
Lasioglossum pauperatum
Megachile argentata
Eucera clypeata
Andrena flavipes
Ceratina dallatorreana
Lasioglossum griseolum
Osmia andrenoides
Anthidium florentinum
Anthidium manicatum
Ceratina cucurbitina
Lasioglossum albocinctum
Heriades rubicola
Ceratina cyanea
Sphecodes gibbus
Osmia caerulescens
Colletes similis
Andrena antonellae
Lasioglossum lativentre
Eucera orianensis
Hoplitis leucomelana
Rhodanthidium septemdentatum
Osmia rufohirta
Hoplitis benoisti
Hoplitis corsaria
Hylaeus taeniolatus
Pseudoanthidium nanum
Heriades crenulata
Halictus scabiosae
Halictus quadricinctus
Pseudoanthidium melanurum
Megachile parietina
Halictus fulvipes
Lasioglossum malachurum
Ceratina parvula
Lasioglossum interruptum
Seladonia gemmea
Megachile albisecta
Osmia leaiana
Andrena cf. niveata
Hylaeus dilatatus
Nomada kholi
Andrena floricola
Sinapis arvensis
Crepis vesicaria
Galactites tomentosus
Raphanus raphanistrum subsp. raphanistrum
Daucus carota
Senecio vulgaris
Hypochaeris achyrophorus
Malva multiflora
Jacobaea delphiniifolia
Sixalix atropurpurea
Cichorium intybus
Jacobaea erucifolia
Sonchus oleraceus
Stachys romana
Heliotropium europaeum
Mentha pulegium subsp. pulegium
Convolvulus arvensis
Pallenis spinosa
Ballota nigra
Borago officinalis
Plantago lagopus
Glebionis coronaria
Trifolium nigrescens
Verbena officinalis
Lotus ornithopodioides
Echium plantagineum
Rubus ulmifolius
Centaurea diluta
Centaurea calcitrapa
Erodium moschatum
Geranium molle
Silene latifolia
Trifolium glomeratum
Bees
Plants
Weight
5 10 15 20 25
Interactions
5 10 15 20 25
B
Borago officinalis
Galactites tomentosus
Glebionis coronaria
Hypochaeris achyrophorus
Jacobaea erucifolia
Sonchus oleraceus
Centaurea calcitrapa
Cichorium intybus
Mentha pulegium subsp. pulegium
Pallenis spinosa
Verbena officinalis
Ballota nigra
Crepis vesicaria
Senecio vulgaris
Malva multiflora
Silene latifolia
Trifolium glomeratum
Daucus carota
Stachys romana
Convolvulus arvensis
Echium plantagineum
Heliotropium europaeum
Plantago lagopus
Sixalix atropurpurea
Centaurea diluta
Erodium moschatum
Lotus ornithopodioides
Trifolium nigrescens
Jacobaea delphiniifolia
Raphanus raphanistrum subsp. raphanistrum
Rubus ulmifolius
Sinapis arvensis
Geranium molle
Andrena hesperia
Andrena minutula
Eucera clypeata
Eucera grisea
Eucera nigrifacies
Eucera orianensis
Heriades rubicola
Nomada femoralis
Osmia scutellaris
Panurgus corsicus
Halictus scabiosae
Megachile albisecta
Osmia leaiana
Rhodanthidium septemdentatum
Seladonia gemmea
Hoplitis leucomelana
Lasioglossum pauperatum
Andrena nigrolivacea
Anthidium florentinum
Anthidium manicatum
Osmia caerulescens
Osmia ligurica
Panurgus dentipes
Andrena floricola
Eucera numida
Andrena colletiformis
Andrena impunctata
Hylaeus brevicornis
Hylaeus clypearis
Lasioglossum pauxillum
Megachile argentata
Megachile melanopyga
Seladonia smaragdula complex
Andrena morio
Bombus hortorum
Bombus terrestris
Ceratina cucurbitina
Ceratina dallatorreana
Heriades truncorum
Hoplitis benoisti
Hoplitis corsaria
Hylaeus taeniolatus
Hylaeus variegatus
Lasioglossum albocinctum
Lasioglossum griseolum
Lasioglossum villosulum
Seladonia subaurata
Trachusa interrupta
Ceratina cyanea
Ceratina parvula
Halictus fulvipes
Halictus quadricinctus
Heriades crenulata
Lasioglossum malachurum
Megachile parietina
Pseudoanthidium melanurum
Andrena cf. niveata
Andrena antonellae
Colletes similis
Lasioglossum lativentre
Osmia andrenoides
Osmia rufohirta
Sphecodes gibbus
Andrena compta
Andrena flavipes
Andrena thoracica
Andrena vetula
Ceratina dentiventris
Lasioglossum interruptum
Pseudoanthidium nanum
Hylaeus dilatatus
Nomada kholi

Figure S5

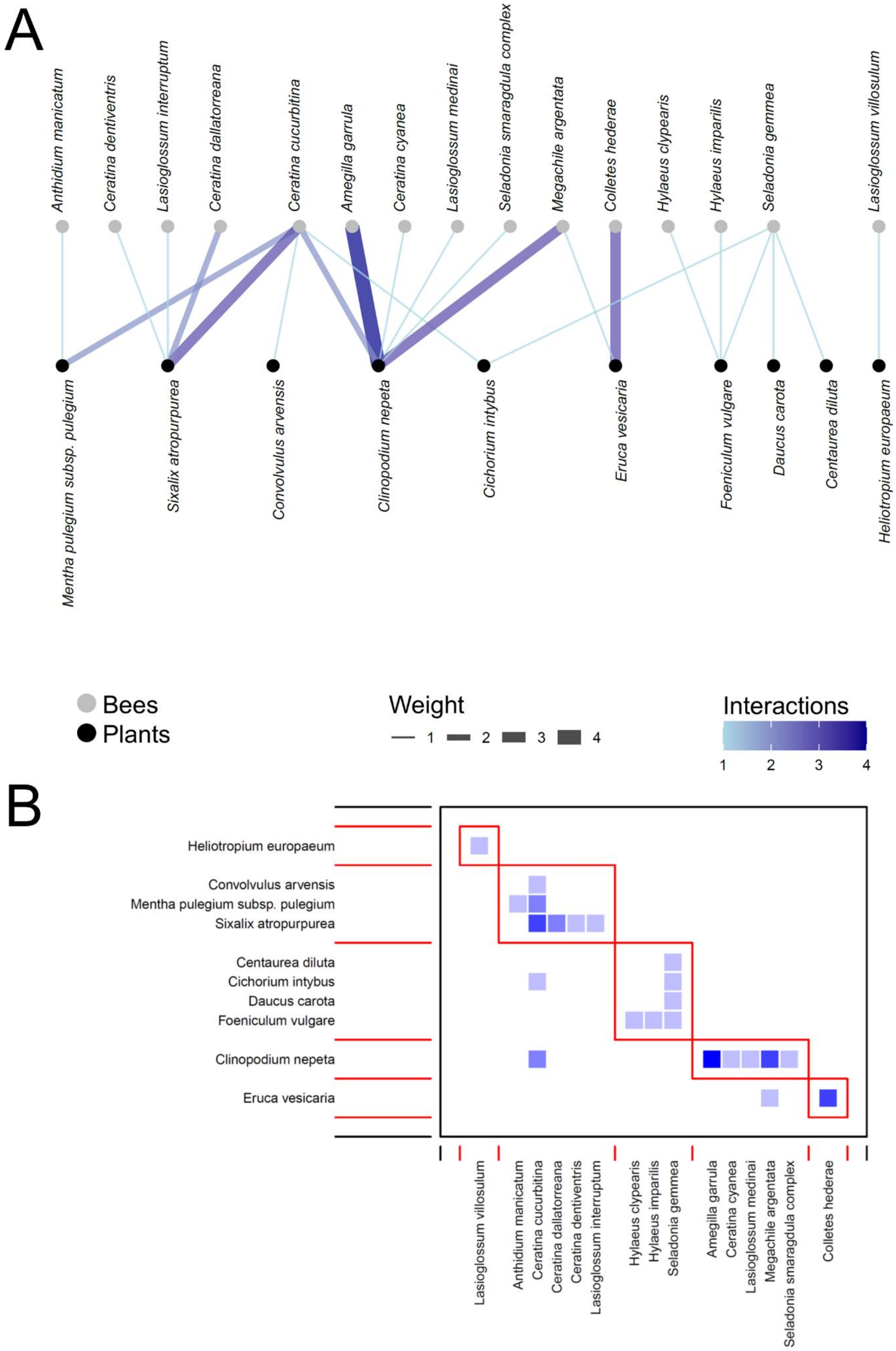
A
Anthidium manicatum
Ceratina dentiventris
Lasioglossum interruptum
Ceratina dallatorreana
Ceratina cucurbitina
Amegilla garrula
Ceratina cyanea
Lasioglossum medinai
Seladonia smaragdula complex
Megachile argentata
Colletes hederae
Hylaeus clypearis
Hylaeus imparilis
Seladonia gemmea
Lasioglossum villosulum
Mentha pulegium subsp. pulegium
Sixalix atropurpurea
Convolvulus arvensis
Clinopodium nepeta
Cichorium intybus
Eruca vesicaria
Foeniculum vulgare
Daucus carota
Centaurea diluta
Heliotropium europaeum
Bees
Plants
Weight
1
2
3
4
Interactions
1
2
3
4
B
Heliotropium europaeum
Convolvulus arvensis
Mentha pulegium subsp. pulegium
Sixalix atropurpurea
Centaurea diluta
Cichorium intybus
Daucus carota
Foeniculum vulgare
Clinopodium nepeta
Eruca vesicaria
Lasioglossum villosulum
Anthidium manicatum
Ceratina cucurbitina
Ceratina dallatorreana
Ceratina dentiventris
Lasioglossum interruptum
Hylaeus clypearis
Hylaeus imparilis
Seladonia gemmea
Amegilla garrula
Ceratina cyanea
Lasioglossum medinai
Megachile argentata
Seladonia smaragdula complex
Colletes hederae

Figure S6

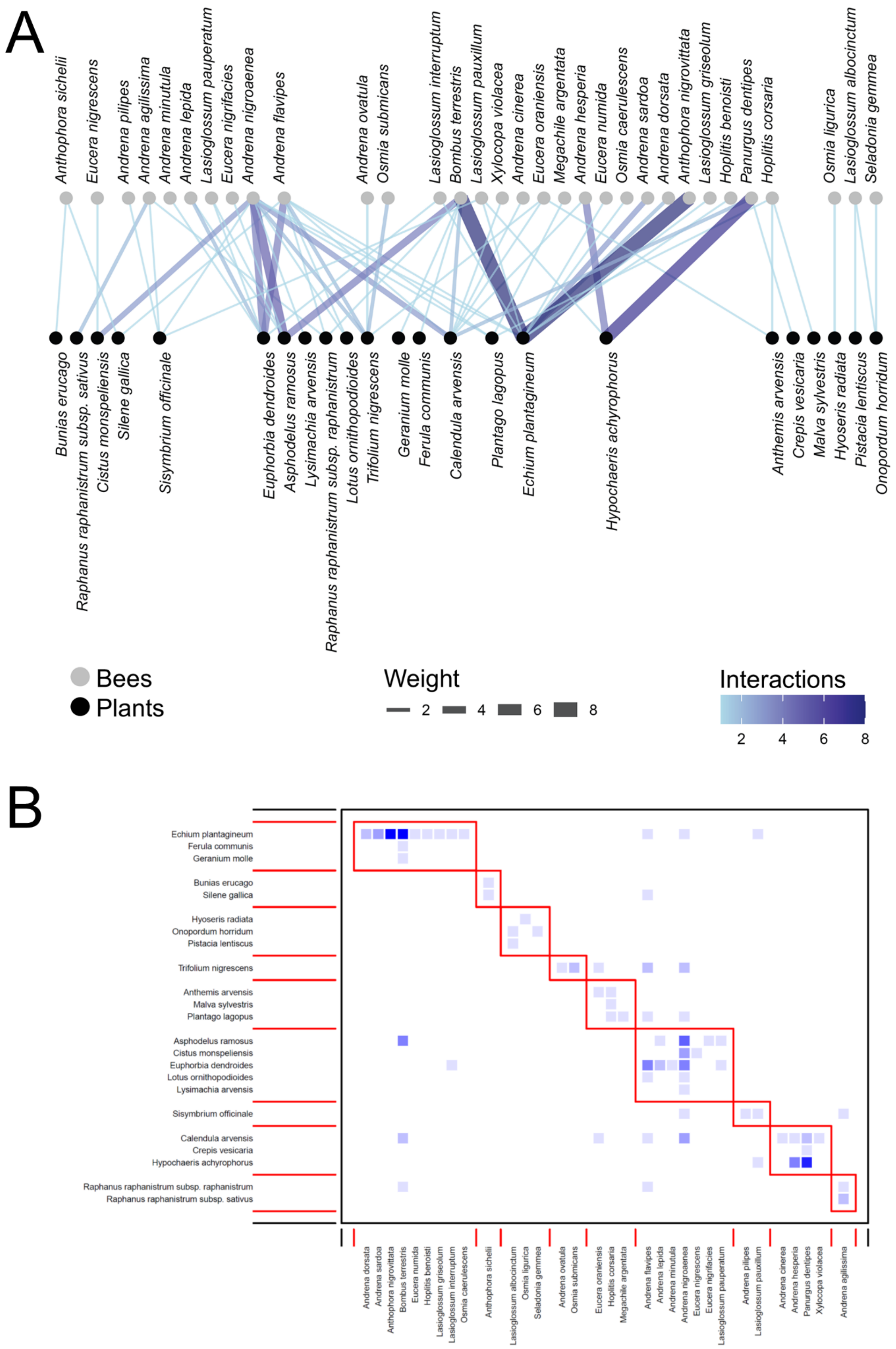

A
Anthophora sichelii
Eucera nigrescens
Andrena pilipes
Andrena agilissima
Andrena minutula
Andrena lepida
Lasioglossum pauperatum
Eucera nigrifacies
Andrena nigroaenea
Andrena flavipes
Andrena ovatula
Osmia submicans
Lasioglossum interruptum
Bombus terrestris
Lasioglossum pauxillum
Xylocopa violacea
Andrena cinerea
Eucera oraniensis
Megachile argentata
Andrena hesperia
Eucera numida
Osmia caerulescens
Andrena sardoa
Andrena dorsata
Anthophora nigrovittata
Lasioglossum griseolum
Hoplitis benoisti
Panurgus dentipes
Hoplitis corsaria
Osmia ligurica
Lasioglossum albocinctum
Seladonia gemmea
Bunias erucago
Raphanus raphanistrum subsp. sativus
Cistus monspeliensis
Silene gallica
Sisymbrium officinale
Euphorbia dendroides
Asphodelus ramosus
Lysimachia arvensis
Raphanus raphanistrum subsp. raphanistrum
Lotus ornithopodioides
Trifolium nigrescens
Geranium molle
Ferula communis
Calendula arvensis
Plantago lagopus
Echium plantagineum
Hypochaeris achyrophorus
Anthemis arvensis
Crepis vesicaria
Malva sylvestris
Hyoseris radiata
Pistacia lentiscus
Onopordum horridum
Bees
Plants
Weight
2
4
6
8
Interactions
2
4
6
8
B
Echium plantagineum
Ferula communis
Geranium molle
Bunias erucago
Silene gallica
Hyoseris radiata
Onopordum horridum
Pistacia lentiscus
Trifolium nigrescens
Anthemis arvensis
Malva sylvestris
Plantago lagopus
Asphodelus ramosus
Cistus monspeliensis
Euphorbia dendroides
Lotus ornithopodioides
Lysimachia arvensis
Sisymbrium officinale
Calendula arvensis
Crepis vesicaria
Hypochaeris achyrophorus
Raphanus raphanistrum subsp. raphanistrum
Raphanus raphanistrum subsp. sativus
Andrena dorsata
Andrena sardoa
Anthophora nigrovittata
Bombus terrestris
Eucera numida
Hoplitis benoisti
Lasioglossum griseolum
Lasioglossum interruptum
Osmia caerulescens
Anthophora sichelii
Lasioglossum albocinctum
Osmia ligurica
Seladonia gemmea
Andrena ovatula
Osmia submicans
Eucera oraniensis
Hoplitis corsaria
Megachile argentata
Andrena flavipes
Andrena lepida
Andrena minutula
Andrena nigroaenea
Eucera nigrescens
Eucera nigrifacies
Lasioglossum pauperatum
Andrena pilipes
Lasioglossum pauxillum
Andrena cinerea
Andrena hesperia
Panurgus dentipes
Xylocopa violacea
Andrena agilissima

Figure S7

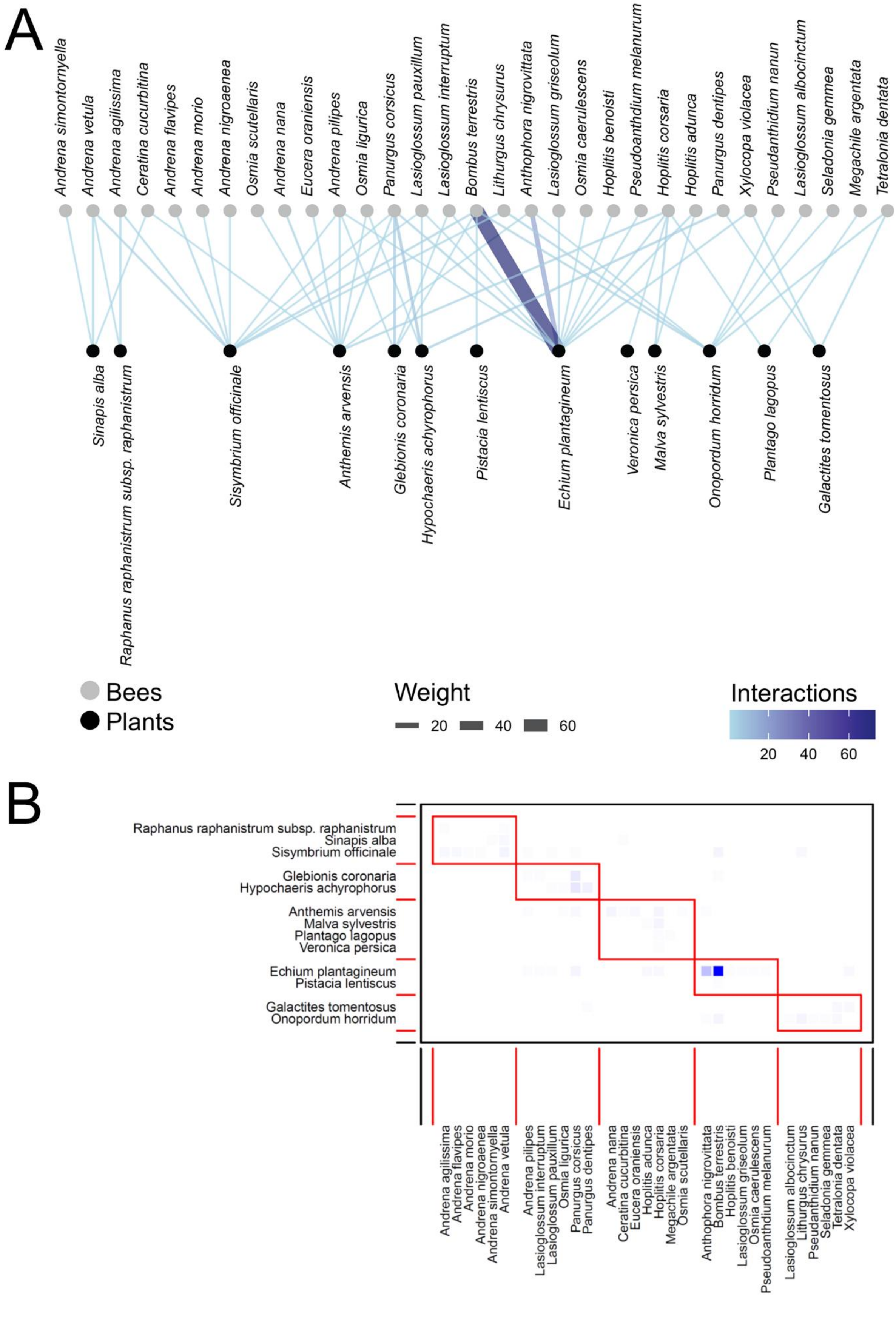
A
Andrena simontornyella
Andrena vetula
Andrena agilissima
Ceratina cucurbitina
Andrena flavipes
Andrena morio
Andrena nigroaenea
Osmia scutellaris
Andrena nana
Eucera oraniensis
Andrena pilipes
Osmia ligurica
Panurgus corsicus
Lasioglossum pauxillum
Lasioglossum interruptum
Bombus terrestris
Lithurgus chrysurus
Anthophora nigrovittata
Lasioglossum griseolum
Osmia caerulescens
Hoplitis benoisti
Pseudoanthidium melanurum
Hoplitis corsaria
Hoplitis adunca
Panurgus dentipes
Xylocopa violacea
Pseudanthidium nanun
Lasioglossum albocinctum
Seladonia gemmea
Megachile argentata
Tetralonia dentata
Sinapis alba
Raphanus raphanistrum subsp. raphanistrum
Sisymbrium officinale
Anthemis arvensis
Glebionis coronaria
Hypochaeris achyrophorus
Pistacia lentiscus
Echium plantagineum
Veronica persica
Malva sylvestris
Onopordum horridum
Plantago lagopus
Galactites tomentosus
Bees
Plants
Weight
20
40
60
Interactions
20
40
60
B
Raphanus raphanistrum subsp. raphanistrum
Sinapis alba
Sisymbrium officinale
Glebionis coronaria
Hypochaeris achyrophorus
Anthemis arvensis
Malva sylvestris
Plantago lagopus
Veronica persica
Echium plantagineum
Pistacia lentiscus
Galactites tomentosus
Onopordum horridum
Andrena agilissima
Andrena flavipes
Andrena morio
Andrena nigroaenea
Andrena simontornyella
Andrena vetula
Andrena pilipes
Lasioglossum interruptum
Lasioglossum pauxillum
Osmia ligurica
Panurgus corsicus
Panurgus dentipes
Andrena nana
Ceratina cucurbitina
Eucera oraniensis
Hoplitis adunca
Hoplitis corsaria
Megachile argentata
Osmia scutellaris
Anthophora nigrovittata
Bombus terrestris
Hoplitis benoisti
Lasioglossum griseolum
Osmia caerulescens
Pseudoanthidium melanurum
Lasioglossum albocinctum
Lithurgus chrysurus
Pseudanthidium nanun
Seladonia gemmea
Tetralonia dentata
Xylocopa violacea

Figure S8

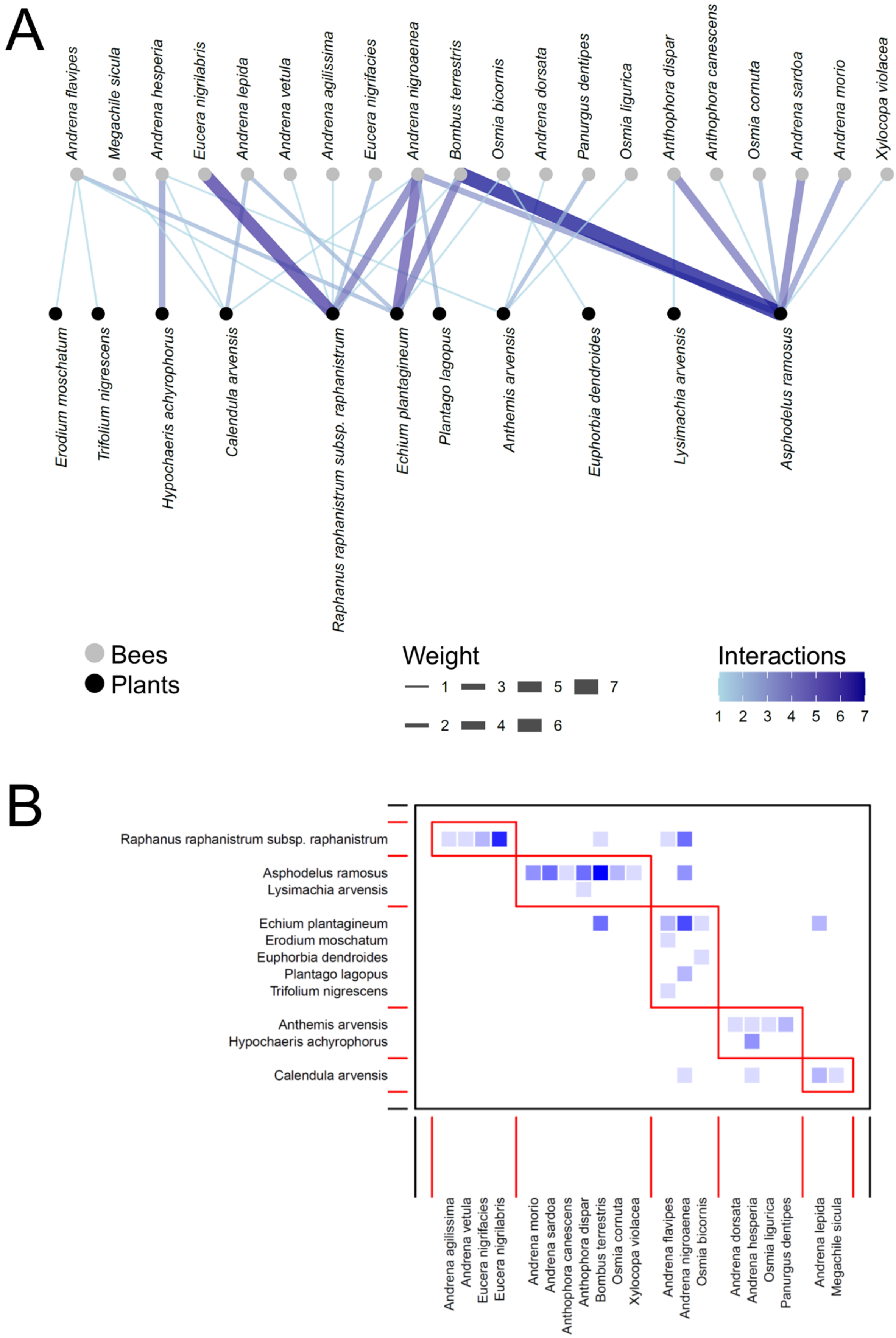
A
Andrena flavipes
Megachile sicula
Andrena hesperia
Eucera nigrilabris
Andrena lepida
Andrena vetula
Andrena agilissima
Eucera nigrifacies
Andrena nigroaenea
Bombus terrestris
Osmia bicornis
Andrena dorsata
Panurgus dentipes
Osmia ligurica
Anthophora dispar
Anthophora canescens
Osmia cornuta
Andrena sardoa
Andrena morio
Xylocopa violacea
Erodium moschatum
Trifolium nigrescens
Hypochaeris achyrophorus
Calendula arvensis
Raphanus raphanistrum subsp. raphanistrum
Echium plantagineum
Plantago lagopus
Anthemis arvensis
Euphorbia dendroides
Lysimachia arvensis
Asphodelus ramosus
Bees
Plants
Weight
1 3 5 7
2 4 6
Interactions
1 2 3 4 5 6 7
B
Raphanus raphanistrum subsp. raphanistrum
Asphodelus ramosus
Lysimachia arvensis
Echium plantagineum
Erodium moschatum
Euphorbia dendroides
Plantago lagopus
Trifolium nigrescens
Anthemis arvensis
Hypochaeris achyrophorus
Calendula arvensis
Andrena agilissima
Andrena vetula
Eucera nigrifacies
Eucera nigrilabris
Andrena morio
Andrena sardoa
Anthophora canescens
Anthophora dispar
Bombus terrestris
Osmia cornuta
Xylocopa violacea
Andrena flavipes
Andrena nigroaenea
Osmia bicornis
Andrena dorsata
Andrena hesperia
Osmia ligurica
Panurgus dentipes
Andrena lepida
Megachile sicula

Figure S9

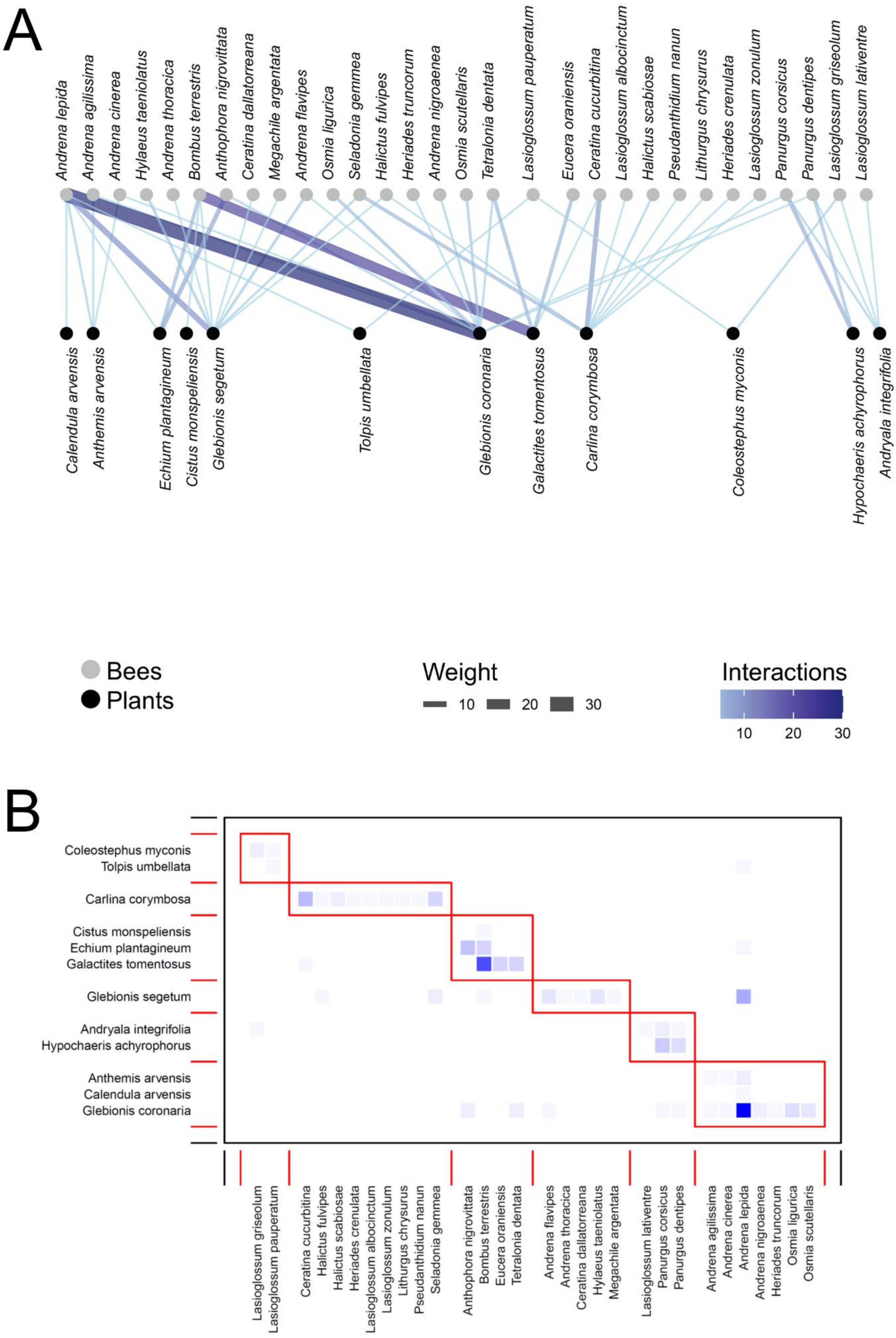

A
Andrena lepida
Andrena agilissima
Andrena cinerea
Hylaeus taeniolatus
Andrena thoracica
Bombus terrestris
Anthophora nigrovittata
Ceratina dallatorreana
Megachile argentata
Andrena flavipes
Osmia ligurica
Seladonia gemmea
Halictus fulvipes
Heriades truncorum
Andrena nigroaenea
Osmia scutellaris
Tetralonia dentata
Lasioglossum pauperatum
Eucera oraniensis
Ceratina cucurbitina
Lasioglossum albocinctum
Halictus scabiosae
Pseudanthidium nanun
Lithurgus chrysurus
Heriades crenulata
Lasioglossum zonulum
Panurgus corsicus
Panurgus dentipes
Lasioglossum griseolum
Lasioglossum lativentre
Calendula arvensis
Anthemis arvensis
Echium plantagineum
Cistus monspeliensis
Glebionis segetum
Tolpis umbellata
Glebionis coronaria
Galactites tomentosus
Carlina corymbosa
Coleostephus myconis
Hypochaeris achyrophorus
Andryala integrifolia
Bees
Plants
Weight
10
20
30
Interactions
10
20
30
B
Coleostephus myconis
Tolpis umbellata
Carlina corymbosa
Cistus monspeliensis
Echium plantagineum
Galactites tomentosus
Glebionis segetum
Andryala integrifolia
Hypochaeris achyrophorus
Anthemis arvensis
Calendula arvensis
Glebionis coronaria
Lasioglossum griseolum
Lasioglossum pauperatum
Ceratina cucurbitina
Halictus fulvipes
Halictus scabiosae
Heriades crenulata
Lasioglossum albocinctum
Lasioglossum zonulum
Lithurgus chrysurus
Pseudanthidium nanun
Seladonia gemmea
Anthophora nigrovittata
Bombus terrestris
Eucera oraniensis
Tetralonia dentata
Andrena flavipes
Andrena thoracica
Ceratina dallatorreana
Hylaeus taeniolatus
Megachile argentata
Lasioglossum lativentre
Panurgus corsicus
Panurgus dentipes
Andrena agilissima
Andrena cinerea
Andrena lepida
Andrena nigroaenea
Heriades truncorum
Osmia ligurica
Osmia scutellaris

Figure S10

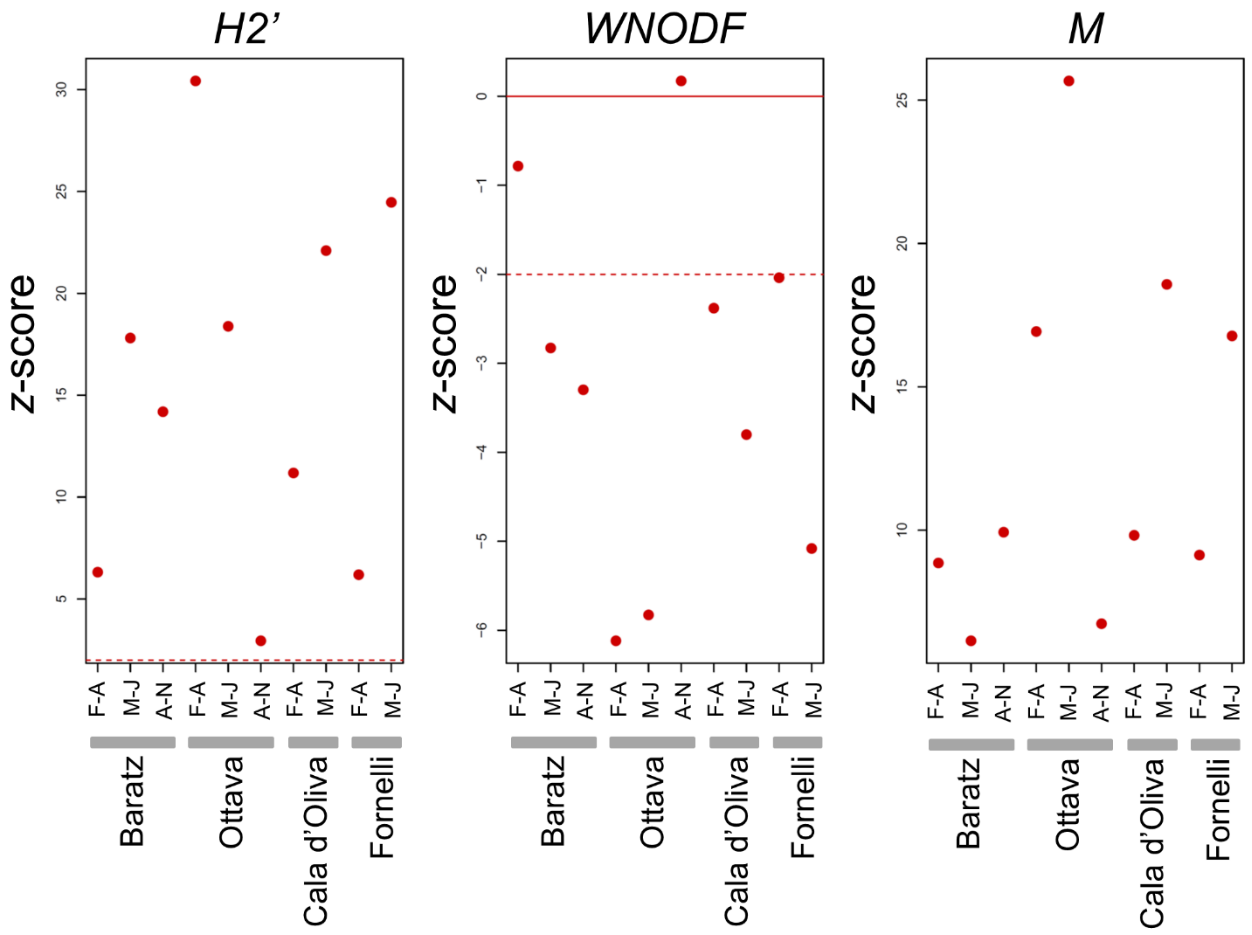
H2'
WNODF
M
z-score
F-A
M-J
A-N
Baratz
Ottava
Cala d'Oliva
Fornelli

Figure S11
